# Toward an Integrated Model of Capsule Regulation in *Cryptococcus neoformans*

**DOI:** 10.1371/journal.ppat.1002411

**Published:** 2011-12-08

**Authors:** Brian C. Haynes, Michael L. Skowyra, Sarah J. Spencer, Stacey R. Gish, Matthew Williams, Elizabeth P. Held, Michael R. Brent, Tamara L. Doering

**Affiliations:** 1 Center for Genome Sciences and Systems Biology and Departments of Computer Science and Genetics, Washington University School of Medicine, St. Louis, Missouri, United States of America; 2 Department of Molecular Microbiology, Washington University School of Medicine, St. Louis, Missouri, United States of America; Carnegie Mellon University, United States of America

## Abstract

*Cryptococcus neoformans* is an opportunistic fungal pathogen that causes serious human disease in immunocompromised populations. Its polysaccharide capsule is a key virulence factor which is regulated in response to growth conditions, becoming enlarged in the context of infection. We used microarray analysis of cells stimulated to form capsule over a range of growth conditions to identify a transcriptional signature associated with capsule enlargement. The signature contains 880 genes, is enriched for genes encoding known capsule regulators, and includes many uncharacterized sequences. One uncharacterized sequence encodes a novel regulator of capsule and of fungal virulence. This factor is a homolog of the yeast protein Ada2, a member of the Spt-Ada-Gcn5 Acetyltransferase (SAGA) complex that regulates transcription of stress response genes via histone acetylation. Consistent with this homology, the *C. neoformans* null mutant exhibits reduced histone H3 lysine 9 acetylation. It is also defective in response to a variety of stress conditions, demonstrating phenotypes that overlap with, but are not identical to, those of other fungi with altered SAGA complexes. The mutant also exhibits significant defects in sexual development and virulence. To establish the role of Ada2 in the broader network of capsule regulation we performed RNA-Seq on strains lacking either Ada2 or one of two other capsule regulators: Cir1 and Nrg1. Analysis of the results suggested that Ada2 functions downstream of both Cir1 and Nrg1 via components of the high osmolarity glycerol (HOG) pathway. To identify direct targets of Ada2, we performed ChIP-Seq analysis of histone acetylation in the Ada2 null mutant. These studies supported the role of Ada2 in the direct regulation of capsule and mating responses and suggested that it may also play a direct role in regulating capsule-independent antiphagocytic virulence factors. These results validate our experimental approach to dissecting capsule regulation and provide multiple targets for future investigation.

## Author Summary

*Cryptococcus neoformans* is a fungal pathogen that causes serious disease in immunocompromised individuals, killing over 600,000 people per year worldwide. A major factor in the ability of this microbe to cause disease is an extensive polysaccharide capsule that surrounds the cell and interferes with the host immune response to infection. This capsule expands dramatically in certain growth conditions, including those found in the mammalian host. We grew cells in multiple conditions and assessed gene expression and capsule size. This allowed us to identify a ‘transcriptional signature’ of genes whose expression correlates with capsule size; we speculated that a subset of these genes acts in capsule regulation. To test this hypothesis, we characterized one previously unstudied gene in this signature and found it to be a novel regulator of capsule expansion, fungal virulence, and mating. This gene encodes cryptococcal Ada2, a well-conserved protein that regulates genes involved in stress response and development. We used phenotypic analysis, RNA sequencing, and chromatin-immunoprecipitation sequencing (ChIP-Seq) to situate Ada2 in the complex network of genes that regulate capsule and other cryptococcal virulence factors. This approach, which yielded insights into the regulation of a critical fungal virulence factor, is applicable to similar questions in other pathogens.

## Introduction

*Cryptococcus neoformans* is an opportunistic fungal pathogen [1]. The disease it causes, cryptococcosis, is contracted by inhalation of infectious particles (spores [2] or dessicated cells), which initiate a pulmonary infection. In the setting of immune compromise the fungus disseminates, with particular predilection for the central nervous system where it can cause a fatal meningoencephalitis. In otherwise healthy hosts, the infection may remain latent for extended periods, emerging in the event of immune compromise [3]. The impact of the disease is significant, especially in populations with limited access to health care, leading to an estimated 600,000 deaths per year [4].

A variety of factors have been implicated in cryptococcal virulence. These include melanin synthesis [5]; urease and phospholipase secretion [6], [7]; titan cell formation [8], [9]; and the ability to survive at host body temperature. Additionally, the main feature that distinguishes *C. neoformans* from other pathogenic fungi is an extensive polysaccharide capsule that surrounds the cell wall and is required for virulence [10]. Capsule size varies tremendously with growth conditions, becoming particularly large during mammalian infection [11]. Capsule expansion can be induced *in vitro* by mimicking aspects of the host environment such as low iron availability, the presence of mammalian serum, and physiological concentrations of carbon dioxide [12]–[14]. Strain virulence correlates with capsule size *in vivo* [15], implicating the regulation of capsule formation as a critical factor in the pathophysiology of cryptococcal disease.

Our current knowledge of capsule regulation derives primarily from studies where mutations of specific genes yield cells with abnormal capsules. A variety of readily assayed phenotypes that are related to the size or nature of the capsule (including cell sedimentation behavior [16], antibody reactivity [17], India ink staining, and colony morphology) has enabled the identification of a wide array of such mutants. Most of these have reduced virulence, emphasizing the central role of the cryptococcal capsule in pathogenesis.

Capsule size is regulated by distinct and overlapping signaling pathways, including those typically associated with stress response. The best-characterized of these, the cAMP pathway, responds to amino acid starvation, low glucose, and elevated carbon dioxide [18]. Stimulation of this pathway leads to high intracellular cAMP levels, which activate the kinase Pka1 [19]. This enzyme in turn activates the C_2_H_2_ zinc finger transcription factor Nrg1, leading to the transcriptional induction of genes that are directly involved in capsule assembly [20]. Pka1 also activates another transcription factor, Rim101, which is necessary for capsule enlargement. Interestingly, activation of Rim101 requires elements of both the cAMP pathway and the pH-responsive Rim signaling pathway [21]. Deletion of the genes encoding Pka1, Nrg1, or Rim101 leads to reduced capsule size.

Iron sensing mechanisms also influence capsule formation. Transcription factors Hap3 and Hap5 are involved in both iron homeostasis and capsule regulation; deletion of the corresponding genes leads to a reduction in capsule size [22]. In addition to Hap3 and Hap5, the iron responsive transcription factor Cir1 also regulates capsule [23], in part by transcriptionally regulating the cAMP pathway. Recently, ChIP-chip studies revealed that Cir1 is directly regulated by another transcription factor, Gat201 [24]. Strains lacking either Cir1 [23] or Gat201 are hypocapsular [25].

Capsule regulation is also influenced by the HOG pathway. Several proteins in this pathway (including Hog1, Pbs2, and Ssk2) negatively regulate capsule size [26]. Epistasis analysis shows that the cAMP pathway is required for this HOG-dependent influence on capsule, but the mechanism of the cross-talk between these two central signaling pathways is unknown. Normal capsule formation also requires proteins in pathways related to temperature sensing [27], sexual development [28], and cell wall integrity [29], [30].

More broadly, chromatin remodeling has been implicated in capsule regulation, by the observation that cells lacking the histone acetyltransferase Gcn5 are hypocapsular [31]. Gcn5 is a member of the well-conserved SAGA complex, which acts in transcriptional regulation from fungi to humans [32]. Sequence analysis suggests that other SAGA proteins are present in *C. neoformans*, but Gcn5 is the best conserved and the only one that has been characterized [31].

Over 60 genes have been identified as important players in capsule formation due to the effects of their deletion on capsule structure or morphology; we refer to such genes as ‘capsule-implicated’ (Table S1). However, because the majority of cryptococcal transcription factors and signaling proteins are uncharacterized, it is likely that important elements of the capsule regulatory network are missing from this group. Furthermore, some capsule-implicated genes may be required for other primary functions, such as cell wall synthesis, that have incidental effects on capsule formation.

As reviewed above, components of several known signaling pathways are required for capsule formation, but there is no model that accounts for the integration of these pathways to regulate capsule growth. To begin constructing such a model, we have identified genes whose RNA levels are correlated with capsule size over a range of *in vitro* conditions. We term this set of genes the transcriptional signature of capsule. This signature includes previously capsule-implicated genes as well as multiple uncharacterized genes encoding putative regulatory factors. We chose to analyze one uncharacterized gene, *ADA2*, which encodes a putative DNA-binding protein. We now show that Ada2 is a novel regulator of capsule and of other virulence-related features of Cryptococcus. Analysis of downstream targets of Ada2 and other capsule regulators by RNA-Seq and ChIP-Seq suggests the context of Ada2 in the capsule regulatory network and illustrates the effectiveness of this approach in unraveling complex regulatory networks.

## Results

### Identifying the Transcriptional Signature of Capsule

**Figure 1:**
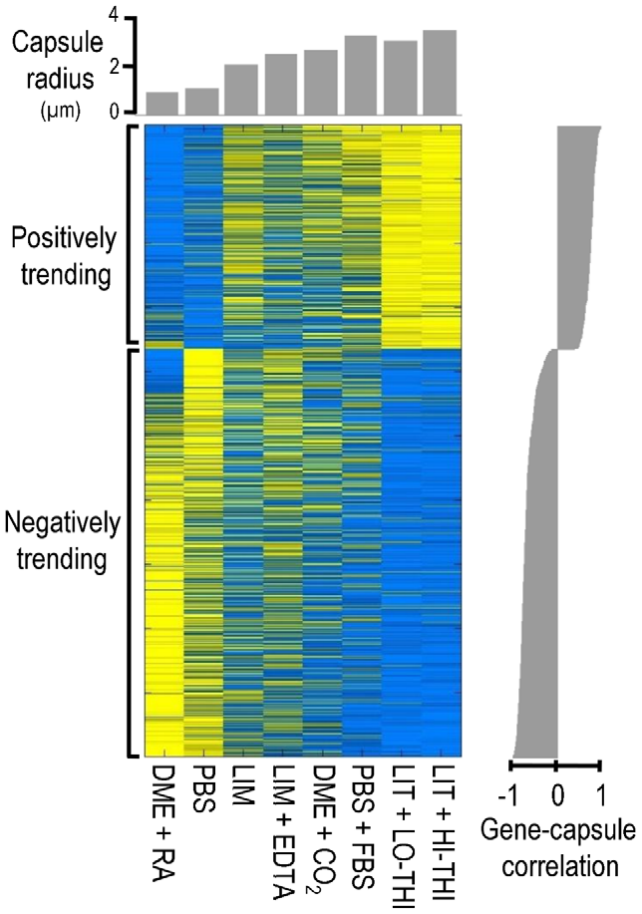
The transcriptional signature of capsule induction. Shown is a heat map of gene expression (blue, low expression; yellow, high expression) for the 880 genes whose expression, as assessed by microarray analysis, trends with capsule size. Cell growth conditions (see Methods) for each column are indicated below the heat map (see Results for abbreviations) and average capsule radius is plotted above (gray bars). The correlation of gene expression and capsule size is plotted at the right.

We reasoned that the transcript abundance of many genes involved in the regulation and synthesis of capsule would correlate with capsule size across multiple growth conditions. To test this hypothesis, and potentially identify capsule regulatory genes beyond those previously reported, we cultured the *C. neoformans* serotype A reference strain H99 in four conditions known to stimulate capsule formation to varying degrees. For each condition, we also cultured the cells in a similar medium that stimulates capsule formation to a lesser extent. The eight conditions used were low iron medium (LIM) with and without the chelating agent ethylenediaminetetraacetic acid (EDTA); phosphate-buffered saline (PBS) with and without fetal bovine serum (FBS); Dulbecco's Modified Eagle's Medium (DMEM) in room air (RA) or in 5% CO_2_; and Littman's medium (LIT) with two concentrations of thiamine (LO-THI / HI-THI). After 24 h the average capsule radius in each culture was assessed by light microscopy. The remaining cells were used to isolate total RNA for hybridization against a *C. neoformans* serotype A/D microarray (http://gtac.wustl.edu/services/microarray/rna-analysis/cryptococcus-neoformans.php). To identify genes whose transcript abundance correlated with capsule size, we compared the transcription profiles over all eight conditions to the quantitative measurements of capsule radius (Figure 1).

Our analysis revealed 880 genes whose transcript abundance correlated significantly with capsule size (Table S2), which we considered the transcriptional signature of capsule induction. Within this set, we identified 316 genes whose transcription correlated positively with capsule radius and 564 genes whose transcription correlated negatively. Among the positively correlated genes, most are involved in responses to stress, including DNA damage repair, trehalose biosynthesis and sugar transport. In contrast, many of the negatively correlated genes are involved in mitochondrial function and ribosome biogenesis.

We expected that some of the genes in the transcriptional signature would specifically influence the formation of capsule (see Discussion). Consistent with this hypothesis, the set of genes whose RNA levels correlated positively with capsule size was enriched for capsule-implicated genes (*p* < 0.02; see Methods); no such enrichment was observed among genes that correlated negatively. Positively correlated genes that are capsule-implicated included the genes encoding regulatory proteins Cir1, Hap5 [22], and Ste20 [33] and the phosphodiesterases Pde1 and Pde2 [34] (see Discussion).

**Figure 2:**
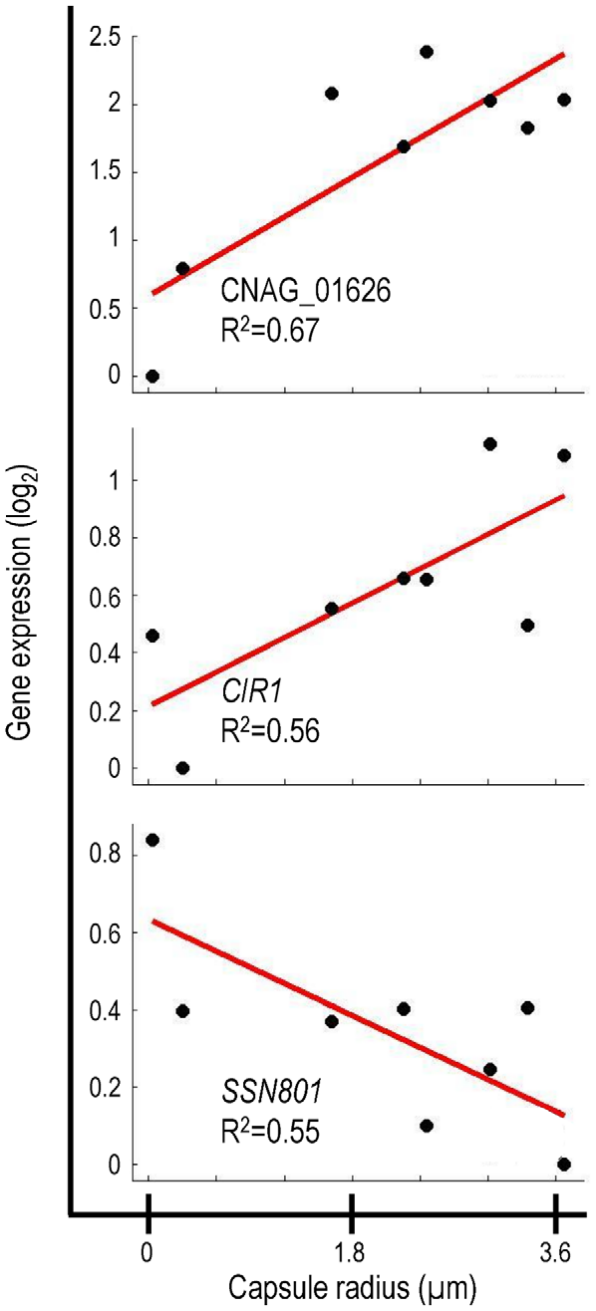
Correlation of gene expression and capsule size for selected genes. Data is shown for three genes that demonstrate correlation between gene expression and capsule size.

The transcriptional signature of capsule included previously uncharacterized genes that encode putative transcription factors, signaling proteins, and sugar transporters (Table S2). It is likely that many of these genes are involved in capsule regulation and assembly. We were particularly interested in one previously uncharacterized gene, CNAG_01626, which encodes a putative DNA binding protein. Expression of CNAG_01626 correlated positively with capsule size (Figure 2). For comparison, Figure 2 also shows the correlations obtained for two cryptococcal transcriptional regulators, *CIR1* and *SSN801*, whose roles in capsule regulation have previously been demonstrated. *CIR1* showed significant positive correlation with capsule size, consistent with the hypocapsular phenotype of *cir1*Δ mutants [23], while *SSN801* exhibited a negative correlation with capsule size, consistent with the hypercapsular phenotype of the corresponding deletion mutant [25].

Given the strong correlation of CNAG_01626 transcription with capsule size, we suspected that the corresponding gene product was a regulator of capsule formation. Because this gene encodes multiple putative DNA-binding domains (Myb-like and SWIRM), we further expected that it might act at a transcriptional level. This hypothesis was supported by the 33% homology we noted between the amino acid sequence predicted for CNAG_01626 and that of the *Saccharomyces cerevisiae* Ada2 protein. In *S. cerevisiae*, Ada2 is a member of the Spt-Ada-Gcn5 Acetyltransferase (SAGA) complex that mediates histone acetylation [35]. Within SAGA, Ada2 is required for proper catalytic activity of the acetyltransferase Gcn5 [36]. Based on the homology between the cryptococcal gene and *S. cerevisiae ADA2*, we decided to refer to CNAG_01626 as *ADA2*.

### Cryptococcal Ada2 Influences Capsule Formation

**Figure 3:**
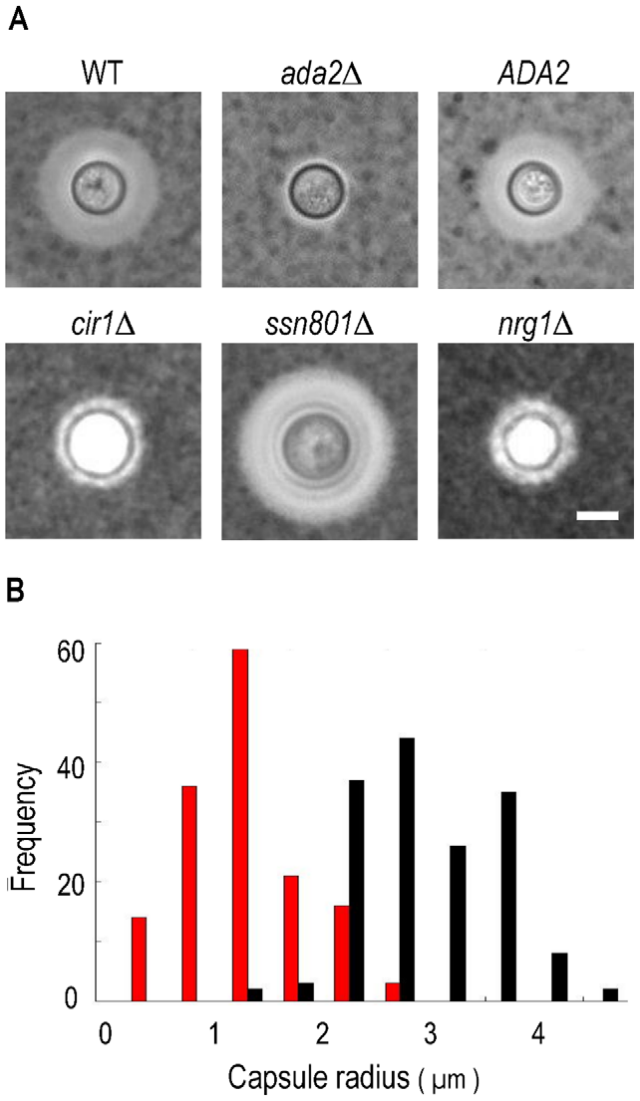
Cells lacking *ADA2* display reduced capsule size under inducing conditions. Panel A, negative staining with India ink of KN99α cells (WT), the indicated deletion strains, and the complemented *ada2*Δ mutant (*ADA2*). All images are at the same magnification. Scale bar, 5 µm. Panel B, histogram of capsule size for the *ada2*Δ mutant (red) and WT (black) populations. Capsule radius is represented in microns.

Since transcription of the cryptococcal *ADA2* gene positively correlates with capsule size, we hypothesized that deleting *ADA2* would yield hypocapsular cells. To assess the role of this putative transcriptional regulator in capsule formation, we replaced the *ADA2* genomic coding sequence with a nourseothricin-resistance marker (*NAT*) in the serotype A strain KN99α, derived from the serotype A reference strain H99 [37]. We then incubated *ada2*Δ mutant cells under capsule-inducing conditions and examined capsule size by negative staining with India ink. Consistent with the microarray analysis (Figure 2), *ada2*Δ mutant cells had dramatically reduced capsule compared to wild type (Figure 3). This phenotype was reversed by complementation with the *ADA2* genomic coding sequence (*ada2*Δ::*ADA2* in Table S3; see Methods).

To facilitate comparison of *ada2*Δ to strains lacking other capsule regulators, we also deleted *CIR1, NRG1,* and *SSN801* in KN99α (see Methods). Consistent with earlier reports, the *ssn801*Δ capsule was enlarged, while the *cir1*Δ and *nrg1*Δ capsules were reduced, similar to the capsule produced by *ada2*Δ (Figure 3, panel A).

### Cryptococcal Ada2 Is Localized to the Nucleus and Is Involved in Histone Acetylation

**Figure 4:**
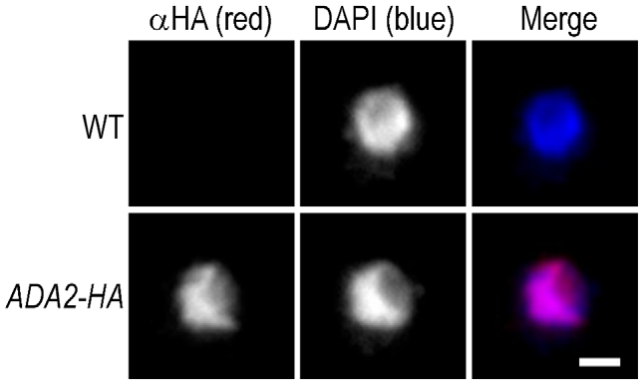
Cryptococcal Ada2 is localized to the nucleus. Wild type cells (WT) and cells modified to express HA epitope-tagged Ada2 from the native locus (*ADA2*-*HA*) were labeled with an antibody against HA (αHA, red), and counter-stained with DAPI (blue) to show the location of chromatin. All images were acquired at the same settings and are shown at the same magnification. Scale bar, 1 µm.

Having demonstrated that cryptococcal Ada2 influences capsule expansion, we proceeded to further investigate its role. Given the function of the SAGA complex in histone acetylation in *S. cerevisiae* [35], we expected that cryptococcal Ada2 would reside in the nucleus. To test our hypothesis, we integrated a hemagglutinin (HA) epitope-tag sequence at the 3′ end of the *ADA2* genomic coding sequence and examined the localization of the tagged protein (Ada2-HA) by immunofluorescence microscopy. Consistent with the nuclear role of Ada2 in *S. cerevisiae*, the tagged cryptococcal protein colocalizes with nuclear DNA (Figure 4).

**Figure 5:**
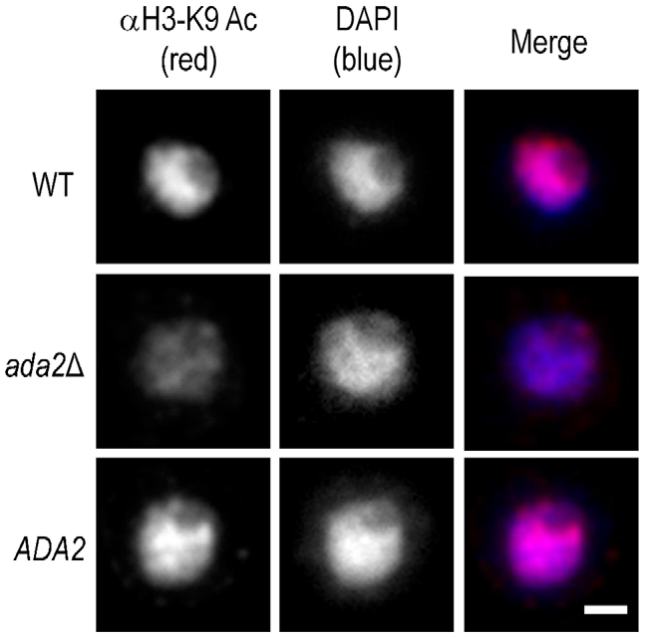
Histone acetylation is markedly reduced in the absence of Ada2. Shown are immunofluorescence micrographs of wild type (WT), *ada2*Δ, and complemented *ada2*Δ (*ADA2*) cells grown in capsule inducing conditions for 90 min and then probed with antibody to H3K9 (αH3-K9). All images were acquired at the same settings and are shown at the same magnification; scale bar, 1 µm.

In *S. cerevisiae*, the SAGA complex activates transcription of stress-responsive genes by acetylating specific lysine residues at the N-terminal tails of histones H2B and H3 [35], [38]. One of these modifications is the acetylation of lysine 9 of histone H3 (H3K9). To assess whether Ada2 is involved in similar histone acetylation in *C. neoformans*, we analyzed the abundance of acetylated H3K9 in the *ada2*Δ mutant by immunofluorescence microscopy using an antibody specific for this modification. We found that the fluorescence intensity of mutant cell nuclei was reduced by at least 50% compared to nuclei of both wild type and complemented cells (Figure 5), a result we confirmed on the population level by immunoblotting with the same antibody (not shown). In contrast, H4 acetylation, which is not SAGA specific [39], [40], showed no difference between the *ada2*Δ mutant and either the wild type or complemented strains (not shown). These results demonstrate the role of CNAG_01626 in histone acetylation, likely in the context of *C. neoformans* SAGA, and strongly support our identification of this novel capsule regulator as the cryptococcal homolog of the *S. cerevisiae ADA2*.

### Ada2 Functions in a Subset of Stress Response Pathways and in Mating

**Figure 6:**
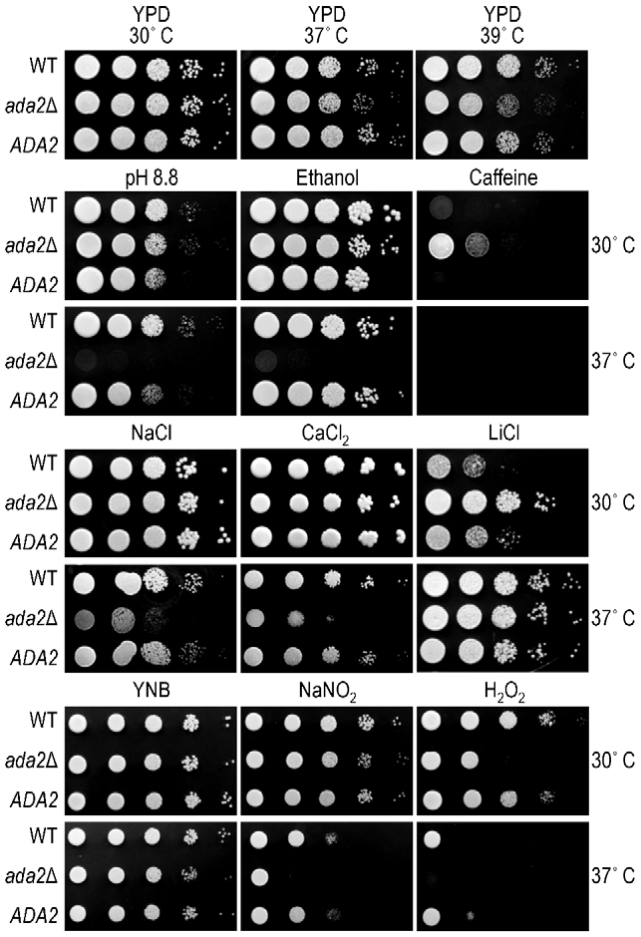
Ada2 is required for growth under certain stress conditions. Ten-fold serial dilutions of the indicated strains were grown in the conditions shown (see Methods for details). Top panel, growth on rich medium (YPD) at the temperatures indicated above the images; middle panels (four rows of images), growth on YPD with the indicated stressor at the temperatures shown at the right; bottom panel (two rows of images), growth on minimal medium (YNB) or YNB with the indicated stressor at the temperatures shown at the right.

In *S. cerevisiae* and other fungi, the SAGA complex regulates the response to stress conditions such as elevated temperature, high salt concentration, and oxidative damage [41], [42]. We found that the *ada2*Δ mutant grew normally compared to wild type on rich medium (YPD) at 30°C (Figure 6). However, the mutant exhibited a subtle growth impairment at 37°C, and a moderate attenuation of growth at 39°C. In all cases, the complemented strain behaved like wild type (Figure 6).

To further compare the phenotype of *ada2*Δ to known fungal SAGA mutants, we next tested a panel of stress conditions for their effect on growth of the wild type, mutant, and complemented strains at both 30 and 37°C (Figure 6). We found that mutant cells were highly sensitive to alkaline pH, with no growth at pH 8.8 at 37°C, while growth at physiological or acidic (5.5) pH was like that of wild type (not shown). Growth of *ada2*Δ at 37°C was also abolished when 6% ethanol was included in the medium, in notable contrast to the growth of wild type cells under this condition, and was impaired at 0.4 M CaCl_2_. Conditions that challenge cell integrity, including media containing calcofluor white (0.2%), congo red (0.5%), low levels of SDS (0.01%), or high sorbitol (2 M), had no effect on mutant growth (not shown). Similarly, KCl (1.2 M) and NaCl (0.4 M) did not alter growth (not shown), although high NaCl concentrations (1.2 M) did reduce growth at 37°C compared to wild type (Figure 6). The *ada2*Δ mutant also showed enhanced growth on caffeine and LiCl at 30°C, although this difference was not observed at the higher temperature tested (see Discussion).

The ability of *C. neoformans* to withstand nitrosative and oxidative stress is required for the virulence of this yeast [43], [44]. We therefore tested the effect of Ada2 absence on cryptococcal sensitivity to compounds that induce such stress. Growth of the *ada2*Δ mutant was not affected by NaNO_2_ (0.5 mM) at 30°C but exhibited a significant defect at 37°C. The mutant was highly sensitive to oxidative stress (0.5 mM H_2_O_2_), with growth attenuated at 30°C and absent at 37°C. (Figure 6). We also examined the ability of this mutant to produce melanin, a feature of *C. neoformans* that is associated with virulence [5]. We observed no difference in melanin production on medium containing L-3,4-dihydroxyphenylalanine (L-DOPA; not shown).

Finally, we tested the sensitivity of the *ada2*Δ strain to several pharmacological agents. These included fluconazole, amphotericin B, and flucytosine, all antifungal compounds used to treat cryptococcal infections. Growth in all cases was comparable to that of wild type, in contrast to the increased fluconazole sensitivity observed upon deletion of *ADA2* in *Candida albicans* [40]. We also tested the sensitivity of *ada2*Δ to FK506, a compound that inhibits calcineurin signaling. A *C. neoformans gcn5*Δ strain has been shown to be FK506 sensitive, suggesting a defect in this pathway [31] (see Discussion); *ada2*Δ cells were even more sensitive to this compound (data not shown).

**Figure 7:**
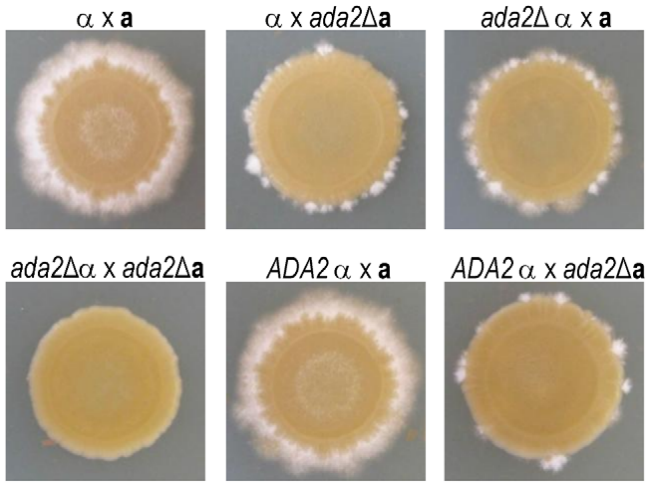
Ada2 is required for normal hyphal development. Wild type (no strain designation), *ada2*Δ, and complemented *ada2*Δ (*ADA2*) strains of opposite mating type were mixed and grown under conditions that induce mating (see Methods). Patches were imaged after 13 days.

While back-crossing the *ada2*Δ mutant, we noticed that this strain was slow to filament. To investigate the potential role of Ada2 in cryptococcal sexual development, we crossed mating type **a** and α cells bearing the *ada2*Δ mutation to KN99**a** and KN99α cells and to each other (Figure 7). Deletion of *ADA2* in either mating type dramatically impaired the formation of dikaryotic filaments in unilateral crosses between the mutant and wild type. A bilateral cross between two *ada2*Δ mutants of opposite mating type showed no visible hyphal development even after 13 days, while the complemented strain behaved identically to wild type.

### Cryptococcal Ada2 is Essential for *C. neoformans* Virulence

**Figure 8:**
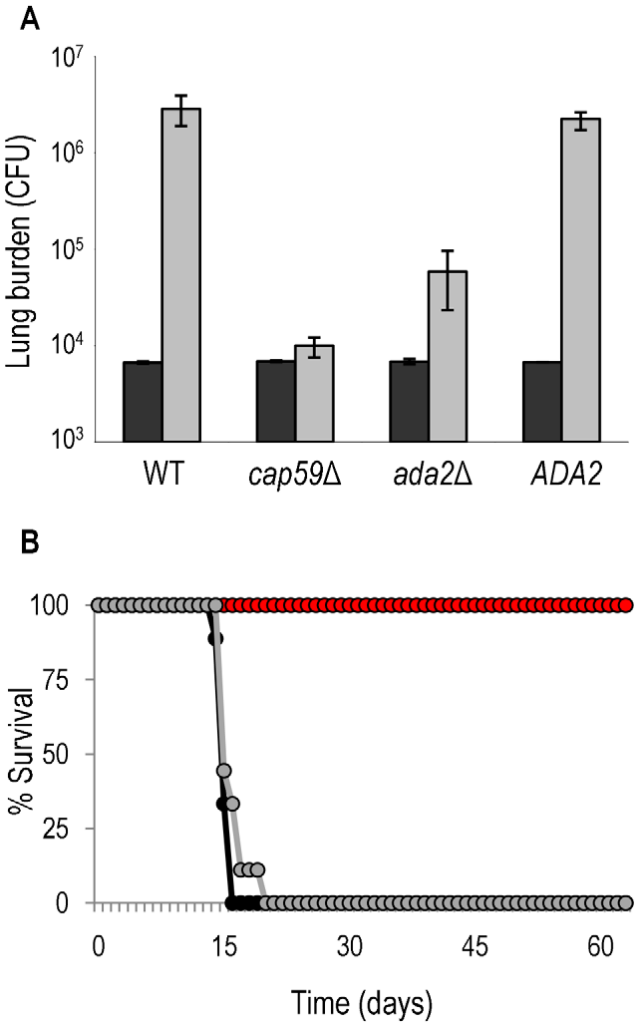
Ada2 is required for growth and virulence in mice. Panel A, C57Bl/6 mice were intranasally inoculated with 1.25 × 10^4^ cells of the indicated strains, and total colony forming units (CFU) were isolated from the lungs after one hour (black bars) or one week (gray bars). The mean±maximum and minimum is shown. Panel B, survival curve of A/Jcr mice that were similarly inoculated with 10^5^ cells of wild type (black), *ada2*Δ (red), or complemented *ada2*Δ (gray). Like those infected with *ada2Δ*, all mice that were infected in the same study with *cap59* survived the entire period (not shown).

The *ada2*Δ mutant displays a smaller capsule, demonstrates reduced resistance to oxidative and nitrosative stress, and grows more slowly at 37°C compared to wild type. Based on these characteristics, we hypothesized that the mutant would also be attenuated for virulence. Indeed, we found that pulmonary growth of the *ada2*Δ mutant was impaired by almost 100-fold compared to the wild type and complemented strains in an inhalational mouse model of cryptococcosis (Figure 8, panel A), although it did grow slightly better than a completely acapsular mutant (*cap59*Δ). To pursue this observation, we conducted a survival study with the same four strains. By three weeks post-inoculation, all mice infected with the wild type and complemented strains had succumbed to the infection (Figure 8, panel B). In contrast, mice infected with the *ada2*Δ or *cap59*Δ mutants remained healthy throughout the study, confirming the requirement for Ada2 in the virulence of this yeast.

### Cryptococcal Ada2 Transcriptionally Regulates Genes Required for Host Adaptation

To identify the genes and processes regulated by Ada2, we used RNA-Seq to perform transcriptome analysis of the *ada2*Δ mutant and wild type cells cultured in either capsule-inducing or capsule non-inducing conditions (see Methods). The majority (92%) of the resulting short reads mapped to the *C. neoformans* serotype A reference sequence [45], indicating the excellent quality of the data. The average 300-fold coverage of the cryptococcal transcripts we obtained in these studies allowed confident sequence identification, and will help improve annotation of the *C. neoformans* genome.

Gene expression analysis revealed 460 genes that were differentially expressed in the *ada2*Δ mutant compared to wild type under the capsule inducing condition; 675 genes were differentially expressed between the two strains under the capsule non-inducing condition (Table S4). We examined the genes whose expression was significantly affected in one or both conditions. Most of these (73%) were regulated in a sign consistent manner in the two conditions (*e.g*. if gene expression was reduced in the *ada2*Δ mutant in non-inducing conditions it was also reduced in the mutant in inducing conditions), although the magnitude of changes did vary. Gene ontology (GO) analysis (see Methods) indicated that processes significantly enriched in the response to loss of Ada2 included ribosomal protein synthesis, sugar transport, and carbohydrate metabolism.

Consistent with the filamentation defect we observed in the *ada2*Δ mutant (Figure 7), we noted several genes downstream of Ada2 that are involved in cryptococcal sexual development (Table 1). Two mating type-specific genes (encoding the homeodomain regulator, Sxi1α, and the pheromone receptor, Ste3α) showed decreased expression in the *ada2*Δ mutant. A variety of genes that are independent of mating type but are implicated in the pheromone response pathway were also found to respond to loss of Ada2 (Table 1).

**Table 1:** Genes downstream of Ada2 implicated in processes related to mating or virulence.

					Fold change (log_2_)	
		Gene Name			*ada2*Δ vs WT	
	Broad ID	*C. neo*	*S. cer*	Description	uninduced	induced
**Mating**	CNAG_06808	*STE3*	*STE3*	pheromone receptor Ste3	**−5.35**	**−7.71**
	CNAG_03137		*SGV1*	Ste11 protein kinase	3.63	**−0.86**
	CNAG_04323		*PRM10*	DUF1212 family protein	**−0.89**	−0.73
	CNAG_06814	*SXI1*		Sxi1	**−0.79**	−0.49
	CNAG_04755		*BCK1*	Ste/Ste11 protein kinase	**−0.59**	−0.23
	CNAG_03706		*GLC7*	phosphatase PP1	**−0.32**	−0.14
	CNAG_02981		*SIN3*	Sin3 protein	**0.47**	0.46
	CNAG_02375		*FIG4*	phosphatase	**0.84**	0.46
	CNAG_05752		*KAR3*	kinesin	**0.81**	0.56
**Capsule**	CNAG_03644	*CAS3*		Cas3	−4.21	**−7.69**
	CNAG_05264	*NSTA*	*YJL216C*	alpha-amylase AmyA	**−2.06**	**−1.97**
	CNAG_03438	*HXT1*	*HXT2*	hexose transporter	**−1.15**	**−1.35**
	CNAG_02797	*CPL1*		pria protein	**−1.01**	−0.65
	CNAG_07937	*CAS1*		O-acetyltransferase	**−0.60**	−0.41
	CNAG_04312	*MAN1*	*PMI40*	mannose-6-phosphate isomerase	**−0.57**	−0.28
	CNAG_07554	*CAP10*		capsule associated protein	**0.43**	−0.15
	CNAG_00124	*CAS32*		Cas32	**−0.50**	0.19
	CNAG_05581	*CHS3*	*CHS3*	chitin synthase 4	0.15	**0.61**
	CNAG_05139	*UGT1*		Ugt1	**0.36**	0.73
	CNAG_02138	*CAS4*	*DNA2*	DNA replication helicase dna2	**0.64**	**1.40**
**Oxidation**	CNAG_05265		*RCK1*	hypothetical protein	2.28	**−5.91**
	CNAG_04415		*YJR096W*	oxidoreductase	**−0.76**	**−3.82**
	CNAG_05027		*FMS1*	amine oxidase	−0.64	**−3.81**
	CNAG_04508		*GRX4*	conserved hypothetical protein	**−2.21**	−3.64
	CNAG_03848	*CNB2310-B*	*GRX7*	glutathione transferase	**−3.21**	**−2.18**
	CNAG_03199		*GRX3*	oxidoreductase superfamily	−0.14	**−1.26**
	CNAG_03936		*PST2*	cytoplasmic protein	**−0.71**	−0.74
	CNAG_01005		*GRX1*	glutathione transferase	**−0.80**	−0.46
	CNAG_00581		*PEP4*	endopeptidase	**−0.30**	−0.44
	CNAG_02859		*POS5*	NADH kinase	**−4.08**	−0.13
**Antiphagocytosis**	CNAG_06762	*GAT204*	*GAT2*	conserved hypothetical protein	−1.28	**−1.44**
	CNAG_06346	*BLP1*		conserved hypothetical protein	−2.70	**−0.86**

Genes listed were identified by differential expression analysis of mutant versus wild type and found to be significantly changed in either capsule non-inducing or inducing conditions. Fold change of mutant versus wild type is indicated in the rightmost columns; bold font indicates statistically significant change.

Our initial interest in Ada2 was stimulated by its importance in capsule synthesis. In the *ada2*Δ mutant, we observed a reduction in transcript abundance for a number of genes that, when deleted, yield small capsules (Table 1). These observations are consistent with the hypocapsular and avirulent phenotypes of the *ada2*Δ mutant. The *ada2*Δ mutant also showed reduced expression for genes involved in oxidative stress; this agrees with the hypersensitivity to oxidative stress observed in the mutant and may also contribute to the avirulent phenotype. Expression of two genes (*BLP1* and *GAT204*), which have recently been implicated in capsule-independent mechanisms of cryptococcal virulence [24], was also reduced in the *ada2*Δ mutant (see Discussion).

### RNA-Seq Analysis Suggests New Relationships in Capsule Regulation

To place Ada2 in the context of the broader capsule regulation network, we performed RNA-Seq analysis on mutants that lack the transcriptional regulators Cir1 and Nrg1. We chose these transcription factors because, like the *ada2*Δ mutant, both the *cir1*Δ and the *nrg1*Δ mutants are hypocapsular, demonstrate attenuated avirulence, and exhibit defects in mating. We identified 1265 genes that were differentially expressed in the *nrg1*Δ mutant compared to wild type under the capsule inducing condition and 1084 under the non-inducing condition (Table S5). For the *cir1*Δ mutant these values were 1257 and 529, respectively (Table S6).

Cryptococcal sexual development is regulated by Cir1 and Nrg1 [23], [20], as well as by Ada2 (Figure 7). To identify common regulatory targets shared by these three transcription factors, we examined the gene expression data from the *ada2*Δ, *cir1*Δ, and *nrg1*Δ mutants. Among genes previously implicated in cryptococcal sexual development, we found that only *SXI1*α was downstream of all three regulators, with its transcription reduced in *nrg1*Δ and *ada2*Δ but increased in *cir1*Δ. Transcription of the pheromone receptor *STE3*α was similarly reduced in *ada2*Δ and elevated in *cir1*Δ although it was not significantly changed in *nrg1*Δ. Genes regulated by Nrg1 included the cell type-specific p21-activated protein kinase *STE20*α, as well as other mating type-independent genes that are involved in sexual development, but these were not regulated by Ada2 or Cir1.

By comparing mutants generated in the same strain background and grown in the same conditions, we were able to confidently identify capsule-implicated genes that are downstream of Cir1 or Nrg1, some of which are also regulated by Ada2. For example, Nrg1 and Ada2 share downstream targets that include *CAS4*, *CAS32*, *CPL1*, *MAN1*, *NSTA*, and *CHS3*. Similarly, *CAP10*, *CAS1*, *CAS4*, and *CPL1* are all downstream of both Cir1 and Ada2. Notably, *CAS4* and *CPL1* are shared targets of all three regulators (Ada2, Nrg1 and Cir1).

In addition to genes that are likely to be directly involved in capsule biosynthesis, we found many genes whose expression was affected by the loss of Cir1 or Nrg1 that are involved in regulating capsule formation. For example, the *nrg1*Δ mutant showed altered transcription of genes in the cAMP pathway, including increased transcription of *RIM101* and decreased transcription of *PKA2* and *PDE2*. Consistent with previous reports [22], we also observed altered transcript levels in the *cir1*Δ mutant that correspond to a number of pH-specific pathway genes, including *RIM9* and *RIM20*. The latter gene product is involved in proteolytic activation of Rim101 [21].

Finally, we discovered that Cir1 and Nrg1 regulate the expression of two HOG pathway genes: absence of either protein led to reduced transcription of *HOG1* and increased transcription of *PBS2*. Additionally, both Cir1 and Nrg1 appeared to enhance the expression of *TUP1* [46], which encodes a regulator that may operate in the HOG pathway. Interestingly, data from a previous microarray study indicated that *ADA2* (at that time uncharacterized) increased in expression upon deletion of HOG pathway members (*HOG1* or *SSK1*) [47] (see Discussion).

### ChIP-Seq Indicates Genes Directly Regulated by Ada2-dependent Histone Acetylation

Ada2 is required for the majority of H3K9 acetylation in *C. neoformans* (Figure 5 and immunoblotting data not shown). We reasoned that localizing Ada2-dependent occurrences of this modification would lead us to genes that are directly regulated by Ada2. We therefore used chromatin immunoprecipitation (ChIP) to isolate DNA directly associated with acetylated H3K9 in *ada2*Δ and wild type cells that we could analyze by short read sequencing (ChIP-Seq).

**Figure 9:**
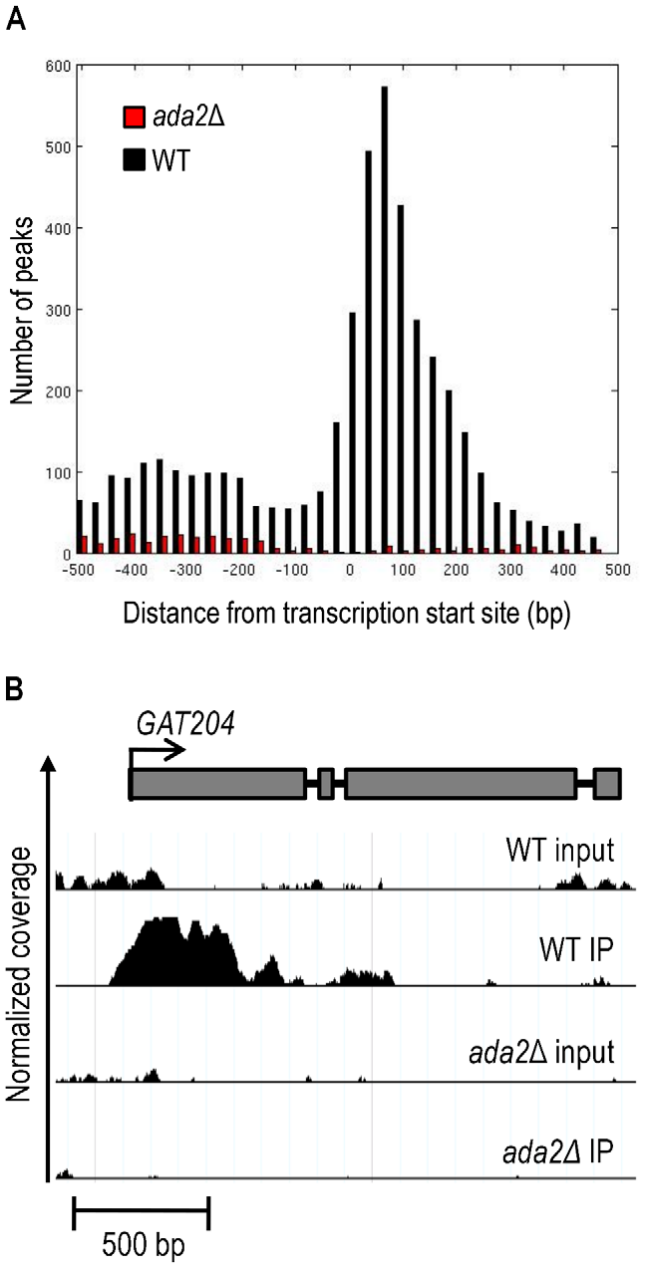
Ada2-dependent acetylation of H3K9 is enriched near gene transcription start sites. ChIP-Seq was performed on wild type (WT) and the *ada2*Δ mutant to identify genes located in the proximity of acetylated H3K9. Panel A, a histogram of peaks that occur within 500 bp of the transcription start site of all identified genes [45]. Panel B, an example of ChIP-Seq data aligned to a gene model of *GAT204*, which was identified as Ada2-dependent by both ChIP-Seq and RNA-Seq. The y-axis represents normalized coverage (reads per million mapped) for samples defined in the text. Coverage is shown for 2 standard deviations above the mean input sample coverage and above. Note that the input DNA profiles are similar for WT and mutant cells, while specific H3K9 associated sequences show TSS-associated peaks only in the WT.

We obtained 84 million short reads from our ChIP-Seq studies, which we aligned to the serotype A reference sequence and analyzed to identify genomic regions with statistically significant coverage (“peaks”) in IP samples compared to input DNA. From triplicate experiments, we identified an average of 2014 peaks in wild type cells, compared to only 364 in *ada2*Δ. This 82% reduction is consistent with our earlier observations on the Ada2-dependence of most H3K9 modification (Figure 5). Consistent with H3K9 acetylation in *S. cerevisiae* [48], [49], the majority of the peaks identified in wild type (75%) were within 500 bp of at least one transcription start site (TSS) as annotated [45] (see Table S7 for a summary of TSS neighboring H3K9 acetylation for wild type or mutant). Most peaks in wild type were also located in the 5′ region immediately downstream of the TSS, with a strong depletion near the TSS and a modest enrichment upstream of the TSS (Figure 9, black bars in panel A). In contrast, only 28% of peaks in *ada2*Δ were within 500 bp of a TSS and almost none of these were downstream of the TSS (Figure 9, red bars in panel A). Thus, not only is histone acetylation in this mutant depleted throughout the genome, the pattern of acetylation is also changed, with the most dramatic depletion occurring in the region immediately downstream of the transcription start site.

The loss of histone acetylation in *ada2*Δ cells suggested Ada2-dependent transcriptional activation at specific loci (see example in Figure 9, panel B). We anticipated that some of these genes would also show reduced transcription by RNA-Seq in the *ada2*Δ mutant; this was indeed the case (p < 0.003). In contrast, we found no such relationship for genes with increased transcription in *ada2*Δ (i.e., genes that are directly or indirectly repressed by Ada2; p > 0.99), consistent with the generally activating function of the SAGA complex. Overall, we found that genes differentially expressed in the *ada2*Δ deletion strain that also lost histone acetylation near the TSS were twice as likely to exhibit reduced transcriptional abundance as genes that did not lose histone acetylation (Figure S1).

We were particularly interested in genes that were activated by Ada2 according to our RNA-Seq analysis and also showed Ada2 dependent H3K9 acetylation in our ChIP-seq analysis. This set is significantly enriched for genes that are directly regulated by Gat201 (p < 0.0001), including *BLP1* and *GAT204* [24]. The genes implicated by both RNA-seq and ChIP-seq also include a number with known capsule phenotypes, such as *CPL1*, *HXT1*, *STE3*α, and *UGT1* (see Discussion).

## Discussion

We analyzed gene expression in *C. neoformans* yeast cells cultured over a diverse set of growth conditions that stimulate capsule production to varying degrees and identified a transcriptional signature of capsule formation. Gene ontology (GO) analysis shows that this signature is enriched for genes involved in stress response, as expected from the conditions we used to induce capsule formation. The signature also contains a significant number of genes that have previously been implicated in capsule regulation; the expression of most of these correlates with capsule in a manner consistent with the null phenotype. The phosphodiesterases Pde1 and Pde2 are exceptions to this pattern: their transcript levels correlated positively with capsule size, while their disruption increases capsule size [34]. Pde1 and Pde2 hydrolyze cAMP to AMP and thereby inhibit the cAMP-dependent activation of regulators known to stimulate capsule formation. Elevated levels of cAMP occurring under capsule inducing conditions may lead to elevated transcription of *PDE*1 and *PDE*2, which would ultimately attenuate the cAMP signal. Feedback inhibition of cAMP signaling via post-translational activation of phosphodiesterases has been documented in both *S. cerevisiae* and *C. neoformans* [50], [34].

One sequence in the transcriptional signature that correlated significantly with capsule size (Figure 2) encoded the putative transcriptional regulator, Ada2. This protein has been characterized most extensively for its role within the SAGA complex, which broadly regulates the transcription of genes involved in stress response and development in multiple organisms [32]. This pattern holds true for *C. neoformans*, based on the increased sensitivity of mutants that lack either *ADA2* (this work) or *GCN5* [31] to reactive oxygen species, ethanol, alkaline pH, elevated temperature, and CaCl_2_ (Figure 6). All of these sensitivities are shared by *S. cerevisiae* SAGA mutants [42], [51], and the last two also are shared by SAGA mutants in other fungi including *C. albicans*, S. *pombe* and *S. kluyveri* [40], [42].

Despite many conserved functions of the SAGA complex across fungal species, several phenotypes of *ada2* mutants in *C. neoformans* differ markedly from those observed in other fungi, perhaps reflecting the specific evolutionary pressures of the cryptococcal niche. Whereas *ada2* mutants in *C. neoformans* display increased caffeine resistance (Figure 6), for example, disruption of SAGA components in *S. cerevisiae*, *S. pombe* and *S. kluyveri* has the opposite effect. Also, *ada2* mutants in *C. neoformans* show an increase in LiCl resistance but no change in KCl resistance (Figure 6), while other fungi defective in SAGA typically exhibit normal growth in LiCl but are KCl sensitive relative to wild type [42]. Interestingly, *ada2* mutants in *C. neoformans* have wild type sensitivity to fluconazole in contrast to *ada2* mutants in *C. albicans*, which have increased sensitivity [40]. Finally, *C. neoformans ada2*Δ differs from other fungi in its regulation of sexual development. In *S. pombe*, the *ada2*Δ mutant is enhanced for mating, probably through a mechanism that does not directly involve histone acetylation [52]. In the *C. neoformans ada2*Δ strain, we instead found dramatically decreased sexual development (Figure 7), reduced transcript abundance of the pheromone receptor *STE3α*, and loss of H3K9 acetylation at the *STE3α* promoter. These results suggest that these two fungi differ in both the direction and the mechanism of Ada2's influence on sexual development.

Recently, another component of the SAGA complex, Gcn5, was shown to play a role in capsule formation and virulence in *C. neoformans* [31]. H99 cells lacking Gcn5, like our mutant lacking Ada2, are hypocapsular and hypovirulent. To compare the roles of these proteins, we examined genes that are differentially expressed by *ada2*Δ and *gcn5*Δ upon growth in DMEM, using our RNA-Seq data for *ada2*Δ (Table S4) and published microarray data sets for *gcn5*Δ [31]. We found a significant overlap in the sets of genes whose expression is affected by each mutation (*p* < 1e-5), supporting the idea that some genes are jointly regulated by Gcn5 and Ada2, probably due to the coordinated role of these proteins in SAGA-mediated histone acetylation.

In addition to shared characteristics, we observed important differences between the *ada2*Δ and *gcn5*Δ mutants at both the phenotypic and transcriptional levels. The *ada2*Δ mutant is more resistant to high temperature, showing ∼10-fold growth inhibition on rich medium at 39°C compared to wild type (Figure 6), a condition where *gcn5*Δ does not grow at all [31]. In contrast, *ada2*Δ is more sensitive than *gcn5*Δ to the calcineurin inhibitor FK506. (The minimal inhibitory concentration (MIC) for *gcn5*Δ is 10-fold below that of its H99 parent [31], while the MIC for *ada2*Δ (performed as in [31]) is at least 67-fold below that of KN99α; data not shown.). We also found that expression of both *STE3*α and *SXI1*α responds to the loss of Ada2 (Table 1), whereas no sexual development genes have been reported to be downstream of Gcn5 [31]. Consistent with this difference, *ada2*Δ is severely defective in filamentation (Figure 7) while *gcn5*Δ filaments normally (T. R. O'Meara and J. A. Alspaugh, personal communication). Furthermore, two genes involved in the recently described ‘antiphagocytic response’ [24], *GAT204* and *BLP1*, showed a loss of both H3K9 acetylation (Figure 9, panel B and Table S7) and expression (Table 1) in *ada2*Δ but no change in expression in *gcn5*Δ [31]. It will be interesting to determine whether these transcriptional differences manifest phenotypically.

The phenotypic differences between *ada2*Δ and *gcn5*Δ may be due to Gcn5-independent functions of Ada2 in *C. neoformans*. Acetylation at some loci may rely on Ada2 partnering with a histone acetyltransferase (HAT) other than Gcn5, or it may be that the regulation of these loci is independent of acetylation altogether. For example, in *S. cerevisiae* Ada2 regulates gene silencing by preventing the spread of repressive chromatin [53]. Such mechanisms remain to be investigated in *C. neoformans*. Given the importance of SAGA in virulence, the roles of Ada2, Gcn5 and other SAGA subunits in *C. neoformans* biology are worthy of further investigation.

After identifying Ada2 as a novel regulator of capsule, we sought to identify elements downstream of it in the capsule regulatory network. To do this, we performed RNA-Seq on the *ada2*Δ mutant and wild type strains, considering genes differentially expressed between these two strains to be downstream of Ada2. To identify probable direct targets of Ada2, we performed ChIP-Seq using antibodies specific for H3K9 acetylation, comparing the *ada2*Δ mutant and wild type strains. We reasoned that genes that lose histone acetylation near their transcription start sites in the *ada2*Δ mutant are likely direct targets of Ada2 via the SAGA complex or another histone acetyltransferase (HAT) complex involving Ada2.

The *ada2*Δ mutant strain revealed a dramatically altered landscape of H3K9 acetylation compared to the wild type, with more than an 80% reduction in acetylated sites across the genome (see Results) and even greater reduction around transcription start sites (Figure 9). This nearly total loss of H3K9 acetylation in the *ada2*Δ mutant is consistent with the established global HAT activity of SAGA in *S. cerevisiae* [54], [55]. In contrast to its broad histone modification activity, SAGA only influences expression of 10% of *S. cerevisiae* genes [39]. RNA-Seq analysis of the *ada2*Δ mutant strain revealed that Ada2 influences transcription of 14% of the genes in *C. neoformans*, indicating that the transcriptional regulatory role of SAGA in *C. neoformans* is also locus specific. ChIP-Seq data further suggest that Ada2 exerts the minority of its influence through direct regulation: only 3% of cryptococcal genes exhibit both altered H3K9 acetylation and expression in *ada2*Δ cells, while 11% exhibit altered expression only. This large indirect response could be mediated in part via the 8 putative transcription factors that Ada2 directly regulates as evidenced by our studies.

Consistent with the activating role of SAGA, the set of genes with reduced expression in *ada2Δ* was significantly enriched for those that lost H3K9 acetylation. (In contrast, genes with increased expression in *ada2Δ* showed no significant overlap with those that lost H3K9 acetylation.) Some genes, including the capsule-implicated gene *UGT1*, showed increased expression together with loss of H3K9 acetylation in the *ada2*Δ mutant, perhaps because H3K9 acetylation at certain loci makes repressor binding sites more accessible. Alternatively, these genes may be directly activated by Ada2 through H3K9 acetylation yet also indirectly repressed by Ada2, which could yield net repression.

We observed phenotypic changes in the *ada2*Δ mutant in sexual development, capsule formation, stress response, and virulence; we also found genes with known roles in these processes to be directly regulated by Ada2 as evidenced by ChIP-Seq and RNA-Seq. For example, we found that Ada2 directly regulates genes encoding proteins implicated in capsule formation, including *HXT1* [56], *CPL1* [25], and *UGT1* [57], consistent with the capsule defect of the *ada2*Δ mutant (Figure 3). We also identified the gene encoding pheromone receptor Ste3 as a direct target of Ada2 in the mating type α (*MAT*α) cells used in these studies, consistent with the observed filamentation defect in *ada2*Δ (Figure 7). Ste3 has also been implicated in mating in *MAT* **a** [28]. Ste3**a** has further been shown to regulate virulence factors including titan cell [8] and capsule formation [28], although no such relation has been reported for Ste3α. If Ada2 also regulates Ste3**a** then it may additionally influence capsule via this pathway in *MAT* **a** cells. Future studies of *MAT* **a** *ada2*Δ mutants will be needed to address this possibility.

Gat201 is a GATA family transcription factor reported to act as a positive regulator of capsule [25]. Interestingly, we observe a significant overlap in the genes that are directly activated by Ada2 (as shown by ChIP-Seq) and those that are direct targets of Gat201 (by ChIP-chip [24]), including the antiphagocytic genes *BLP1* and *GAT204*. Since the SAGA complex typically works in concert with other transcription factors, this suggests that Ada2 may work with Gat201 to activate transcription. It may be that Gat201 recruits Ada2 in the context of SAGA for these purposes. Alternatively, another factor may recruit the SAGA complex, which then enables Gat201 to bind.

**Figure 10:**
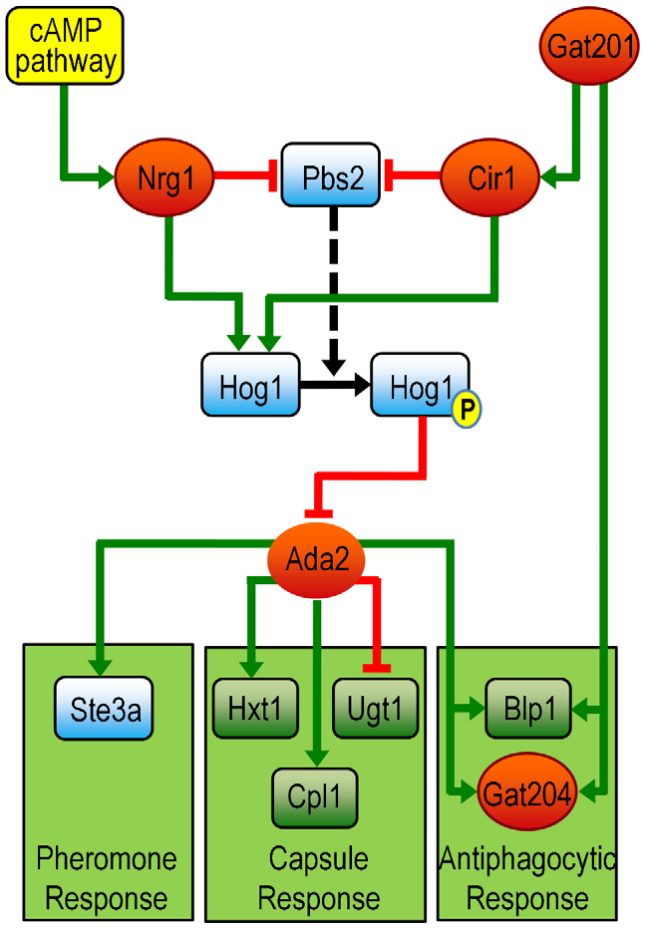
A model of Ada2 within the broader network of capsule, mating, and antiphagocytic responses (see text for details). Links from Cir1 and Nrg1 are supported by RNA-Seq data presented here. Links from Hog1 and Pbs2 are supported by published microarray data [47]; links from Gat201 are supported by published data from microarrays and ChIP-chip [24]; and links from Ada2 are supported by RNA-Seq and ChIP-chip data presented here. Red ovals, transcription factors; blue rounded rectangles, signaling proteins; green rounded rectangles, other proteins; green lines, stimulation of transcription; red lines, inhibition of transcription; solid black arrow, phosphorylation; P, phosphate; dashed arrow, catalysis by Pbs2.

To explore the interplay between regulatory pathways we considered two transcription factors, Cir1 [23] and Nrg1 [20], which like Ada2 enhance both capsule and mating responses. In the set of genes regulated by Cir1 and Nrg1 (Table S5 and Table S6), we identified two that encode proteins in the HOG pathway, Hog1 and Pbs2; both Cir1 and Nrg1 transcriptionally repress Pbs2 and activate Hog1. Cells lacking either Pbs2 or Hog1 show increased capsule formation and sexual development [26]. Furthermore, both *ADA2* and *GCN5* were shown in earlier work to be transcriptionally repressed by Hog1 under nutrient rich conditions [47]. This observation, in conjunction with our data, suggests that the HOG pathway may regulate capsule and mating via Ada2 (Figure 10). Our transcriptional analysis suggests that Nrg1 and Cir1 operate on the HOG pathway through a shared incoherent feed-forward loop, by transcriptionally activating Hog1 and simultaneously repressing Pbs2. In nutrient rich conditions, Hog1 is constitutively phosphorylated by Pbs2 and represses mating and capsule. The logic of this circuit implies that in capsule inducing conditions Cir1 and Nrg1 repress transcription of *PBS2*; this leads to reduced levels of phosphorylated Hog1, thus derepressing *ADA2* transcription and enhancing capsule formation. Simultaneously transcription of *HOG1* is increased, leading to an even greater abundance of unphosphorylated Hog1. This increase in Pbs2 substrate may allow rapid restoration of the transcriptional repression of Ada2 once the environmental cues for capsule induction are no longer present. Although transcript levels of *ADA2* were not significantly altered in the *nrg1*Δ and *cir1*Δ mutants at the 90-minute time point that we tested, *ADA2* expression may be affected by these mutations at later time points. Future studies will also be needed to determine whether the influences of Cir1 and Nrg1 on *PBS2* and *HOG1* result from direct or indirect regulation, and to better characterize the exact structure and function of this hypothesized regulatory circuit. This model, rich in testable hypotheses, illustrates the power of combining RNA-Seq and ChIP-Seq data in an integrated analysis.

Our identification of Ada2 in the capsule transcriptional network validates our strategy for probing capsule regulation and suggests that it may be valuable in studying the regulation of other processes that are important in microbial pathogenesis. These studies also lead in numerous exciting directions for the future. Our parallel comparison of multiple mutants in the same strain background and growth conditions has allowed us to identify previously unobserved relationships among capsule regulators, which we look forward to testing. Our analysis of the transcriptional signature of capsule induction also suggests multiple potential transcription factors that can be pursued to further probe the complex confluence of pathways that lead to capsule synthesis, and our implementation of ChIP-Seq in *C. neoformans* demonstrates a high-resolution way for differentiating direct from indirect regulatory relationships. Overall, our work highlights the power of integrative transcriptome analysis to dissect regulatory networks in *C. neoformans* and beyond.

## Materials and Methods

### Ethics Statement

All animal studies were reviewed and approved by the Animal Studies Committee of Washington University School of Medicine and conducted according to the National Institutes of Health guidelines for housing and care of laboratory animals.

### Materials

All chemicals were from Sigma, primers were from Invitrogen, and restriction enzymes were from New England Biolabs unless otherwise noted. All kits and enzymes were used according to manufacturer recommendations unless otherwise specified.

### Strains and Growth Conditions

All strains used in this study are capsule serotype A, which causes the majority of illness in immunocompromised patients [58], and are listed in Table S3. Microarray experiments to identify the transcriptional signature of capsule were performed with *C. neoformans* H99 and mutants were constructed in *C. neoformans* KN99. All cells were grown with continuous shaking (230 rpm) at 30°C in YPD medium (1% w/v yeast extract, 2% w/v peptone, 2% w/v glucose), or at 30°C on agar plates (YPD medium with 2% w/v agar). As appropriate, media were supplemented with either 100 µg/ml of nourseothricin (from Werner BioAgents) or 100 µg/ml of Geneticin (G418; from Invitrogen). Genetic crosses were performed at room temperature (RT) in the dark on V8 agar plates (5% v/v V8 juice, 0.05% w/v KH_2_PO_4_ pH 5, 4% w/v agar) as described [37]. To induce expression of genes involved in capsule formation, cells cultured overnight in YPD were collected by centrifugation, washed in DMEM, and adjusted to 4 × 10^7^ cells/ml in DMEM. This cell suspension was first incubated at 30°C in room air for 2 hr, then shifted to 37°C with 5% CO_2_ for 1.5 hr. Conditions used for phenotypic testing of mutants are detailed in Text S1.

### RNA Isolation

Approximately 2 × 10^8^ cells were collected by centrifugation, suspended in TRIzol reagent (from Invitrogen), and subjected to mechanical lysis by bead beating at 4°C with 0.5-mm glass beads for 1 min, followed by a 2-min rest, for a total of 5 cycles. Following lysis, total RNA was extracted according to the manufacturer's instructions. Residual DNA was removed from the RNA preparation by treatment with the Turbo DNA-free kit (from Ambion) according to the manufacturer's instructions.

### Microarray Experiments

H99 cells were cultured overnight at 37°C in the following eight conditions: low iron medium with or without both 500 mM ethylenediaminetetraacetic acid (EDTA) and 10 mM bathophenanthroline disulfonate (BPDS); phosphate-buffered saline (PBS) with or without 10% v/v fetal bovine serum; Dulbecco's Modified Eagle's Medium (from Sigma) in room air or 5% CO_2_; and Littman's medium [13] with either 0.01 µg/ml or 1 µg/ml thiamine. All experiments were performed in triplicate. Total RNA was isolated from each culture and hybridized to a *C. neoformans* serotype A/D microarray against a shared reference pool of RNA as described [59]. Slides were scanned on a Perkin-Elmer ScanArray Express HT scanner to measure Cy3 and Cy5 fluorescence as described [59]. Normalization of the raw spot intensities was performed using LIMMA [60]. Normalization was performed using normexp with an offset of 50 followed by Loess and values for replicate probes on the array were averaged to represent expression of the associated gene. The correlation between gene expression and capsule radius (which was measured for each sample at the time of RNA isolation) was assessed using SAM [61] and statistical significance was calculated using a false discovery threshold of 5%. A hypergeometric test was applied to determine the enrichment of capsule-implicated genes (genes whose mutation yields an alteration in capsule size or morphology; Table S1) in the positively and negatively correlating sets of genes. The complete array data set is available at GEO accession number GSE31911.

### Strain Construction

The *C. neoformans* H99 reference sequence was accessed through the Fungal Genome Initiative database at the Broad Institute of MIT and Harvard available at <http://www.broadinstitute.org/science/projects/projects>. Cryptococcal genomic DNA was isolated as described [62] and a split-marker approach [63] was used to replace each genomic coding sequence of interest with a nourseothricin resistance marker (*NAT*) by homologous recombination. Each mutant was also labeled with a unique signature tag by incorporating a 13-bp tag sequence (see Table S8) and an 18-bp priming site (5′ - AGAGACCTCGTGGACATC - 3′) immediately downstream of *NAT*. We also used the split-marker gene replacement approach to introduce a single copy of the hemagglutinin (HA) epitope-tag sequence at the 3′ end of the *ADA2* genomic coding sequence. Details of strain construction are provided in Text S1, Table S9 and Table S10.

### Capsule Induction and Quantitation of Capsule Size

Cells cultured in YPD were washed extensively in DMEM, then adjusted to 10^6^ cells/ml in DMEM and incubated for 24 hours at 37°C with 5% CO_2_. Capsules were visualized by negative staining with India ink, and a minimum of 100 randomly chosen cells were imaged with identical acquisition settings on a Zeiss Axioskop 2 MOT Plus wide-field fluorescence microscope. Capsule radius was calculated as half the difference between the capsule diameter and the diameter of the cell body.

### Immunofluorescence Microscopy

Cells were cultured overnight in YPD, and the expression of genes involved in capsule formation was induced as described above. Cells were then collected by centrifugation, washed in PBS, adjusted to 3 × 10^8^ cells/ml in 4% w/v formaldehyde buffered in PBS, and incubated for 1 hr with rotation. Fixed cells were collected by centrifugation (1 min, 400 × g), washed extensively in PBS, adjusted to 3 × 10^8^ cells/ml in Lysis Buffer (50 mM sodium citrate pH 6.0, 1 M sorbitol, 35 mM β-mercaptoethanol) plus 25 mg/ml Lysing Enzymes (from Trichoderma harzianum), and incubated for 1 hr at 30°C. Digested cells were collected by centrifugation (3 min, 400 × g), washed with HS Buffer (100 mM HEPES pH 7.5, 1 M sorbitol), and resuspended in 100-200 µl of HS Buffer. The cell suspension was spotted in 20-µl aliquots on a glass microscope slide coated with 0.1% w/v poly-L-lysine and incubated for 20 min at RT.

All subsequent treatments and washes were performed by the application of 20-µl volumes and incubation at RT, and were followed by aspiration. The slides were first treated with HS Buffer containing 1% v/v Triton X-100 and incubated for 10 min; they were then washed with PBS and treated with Blocking Buffer (5% v/v goat serum, 0.02% v/v Tween-20 in PBS) for 1 hr. Cells were next labeled with either a high-affinity rat anti-HA monoclonal antibody (0.2 µg/ml in Blocking Buffer; from Roche), a rabbit anti-acetyl-Histone H3 polyclonal antibody (0.5 µg/ml in Blocking Buffer; from Millipore), a rabbit anti-acetyl-Histone H4 polyclonal antibody (1 µg/ml in Blocking Buffer; from Millipore), or Blocking Buffer alone overnight in a moist chamber at 4°C. Cells were then washed with Blocking Buffer, and treated for 1 hr in the dark with either Alexa Fluor 594 goat anti-rat IgG or Alexa Fluor 594 goat anti-rabbit IgG (2 µg/ml in Blocking Buffer; from Invitrogen). Next, cells were again washed with Blocking Buffer, counterstained with 4′,6-diamidino-2-phenylindole (DAPI; 5 µg/ml in PBS) for 20 min in the dark, washed with PBS, allowed to air-dry, and mounted in Prolong Gold (from Invitrogen). Brightfield and fluorescence images were acquired simultaneously on a Zeiss Axioskop 2 MOT Plus wide-field fluorescence microscope. All samples were imaged with identical acquisition settings.

### Growth and Virulence in Mice

Two types of animal studies were performed, both in compliance with all institutional guidelines for animal experimentation. For a short term model of fungal survival in the mouse lung, strains to be tested were cultured overnight in YPD medium, collected by centrifugation, washed in PBS, and diluted to 2.5 × 10^5^ cells/ml in PBS. For each strain, eight 4–6 week-old female C57Bl/6 mice (from Jackson Laboratories) were anesthetized with a combination of ketaset-HCl and xylazine, and inoculated intranasally with 50 µl of the prepared yeast suspension. Three animals from each cohort were sacrificed at 1 hr post-inoculation; the remaining five were sacrificed after 7 days. Lungs were harvested following sacrifice, and homogenized in PBS. Serial dilutions of the homogenate were plated on YPD agar for determination of colony-forming units (CFU). Initial inocula were also plated to confirm CFU.

To assess longer-term affects of cryptococcal infection, each strain was cultured and prepared as above, with the exception that the cells were diluted to 2 × 10^6^ cells/ml in PBS. Ten 4–6 week-old female A/Jcr mice (from the National Cancer Institute) were anesthetized as described above and inoculated intranasally with 100 µl of the prepared cell suspension. The animals were weighed within 1 hr post-inoculation, and subsequently on every other day. Mice were sacrificed if weight decreased to a value less than 80% of peak weight (an outcome which in this protocol precedes any signs of disease) or upon completion of the study. Initial inocula were plated to confirm CFUs.

### RNA-Seq

Cells were cultured overnight in YPD, and grown for 90 minutes in either capsule-inducing (DMEM, 37°C, 5% CO_2_) or capsule non-inducing (DMEM, 30°C, room air) conditions prior to isolation of total RNA. A minimum of two biological replicates were performed for each mutant (*ada2*Δ, *nrg1*Δ and *cir1*Δ) and four for wild type. PolyA+ RNA was purified from total RNA using the Dynabeads mRNA Purification Kit according to the manufacturer's instructions (from Invitrogen). Each sample was resuspended in 2 µl of 100 mM zinc acetate and heated at 60°C for 3 minutes to fragment the RNA by hydrolysis. The reaction was quenched by the addition of 2 µl volumes of 200 mM EDTA and purified with an Illustra Microspin G25 column (from GE Healthcare). First strand cDNA was made using hexameric random primers and SuperScript III Reverse Transcriptase (from Invitrogen) according to the manufacturer recommendations, and the product was treated with *E. coli* DNA ligase, DNA polymerase I, and RNase H to prepare double stranded cDNA using standard methods. The cDNA libraries were end-repaired with a Quick Blunting kit (from New England BioLabs) and A-tailed using Klenow exo- and dATP. Illumina adapters with four base barcodes were ligated to the cDNA and fragments ranging from 150-250 bp in size were selected using gel electrophoresis as recommended by the manufacturer. The libraries were enriched in a 10-cycle PCR with Phusion Hot Start II High-Fidelity DNA Polymerase (from Finnzymes Reagents) and pooled in equimolar ratios for multiplex sequencing. Single read, 36-cycle runs were completed on the Illumina Genome Analyzer IIx.

Sequenced reads were aligned to the *C. neoformans* H99 reference sequence [45] using Tophat [64]. Reads that aligned uniquely to the reference sequence were considered for gene expression quantification with Cufflinks [65] using the current genome annotation provided by the Broad institute. Gene expression was normalized using the Cufflinks provided option for quartile normalization. Differential expression analysis comparing mutant to wild type was performed with LIMMA [60] and ELNN [66] using a 5% false discovery rate. Genes whose expression was found to be significantly changed by either analysis method were counted as differentially expressed. RNA-Seq data is available at GEO accession number GSE32049.

### Gene Ontology (GO) Enrichment

GO enrichment analysis was performed by assigning GO categories to each gene according to the Broad Institute's PFAM annotations using the mapping provided by the Gene Ontology project (http://www.geneontology.org/external2go/pfam2go). A hypergeometric test was applied for each GO category, the resulting p-values were corrected for multiple hypothesis testing, and a cutoff of 0.05 was used to determine significance.

### Chromatin Immunoprecipitation (ChIP)

Wild type and *ada2*Δ cells were cultured in triplicate overnight in YPD, and grown in capsule-inducing conditions (DMEM, 37 °C, 5% CO_2_) for 90 minutes. Cells were then fixed for 5 min in 1% (v/v) formaldehyde, and the reaction quenched with a final concentration of 125 mM glycine. Fixed cells were collected by centrifugation, washed with PBS, and resuspended in Buffer A (50 mM HEPES pH 7.5, 140 mM NaCl, 1 mM EDTA, 1% v/v Triton X-100, 0.1% w/v sodium deoxycholate) supplemented with protease inhibitors and 20 mM sodium butyrate (a histone deacetylase inhibitor). The cell suspension was subjected to mechanical bead-beating with 0.5-mm zirconium silicate beads for 2 min at 4°C, followed by a 2-min rest, for a total of 10 cycles. Chromatin was then sheared by sonicating the lysate for 30 sec at 40% power output, followed by a 1-min rest on ice, for a total of 40 cycles, and the lysate clarified by centrifugation. A fraction of the sheared chromatin was reserved as an input sample and the remainder was used for immunoprecipitation. Acetylated histone H3 was immunoprecipitated overnight with anti-acetyl-H3 (K9) antibody (from Millipore) tethered to protein-A sepharose (10 ml in a total volume of 700 ml). The beads were next washed sequentially in Buffer A, Buffer B (50 mM HEPES pH 7.5, 500 mM NaCl, 1 mM EDTA, 1% (v/v) Triton X-100, 0.1% (w/v) sodium deoxycholate), Buffer C (10 mM Tris-HCl pH 8.0, 250 mM LiCl, 1 mM EDTA, 0.5% (v/v) NP-40, 0.5% w/v sodium deoxycholate), and Buffer D (10 mM Tris, 1 mM EDTA), and immunoprecipitated protein was eluted with Buffer E (50 mM Tris pH 8.0, 10 mM EDTA, 1% (w/v) SDS). Crosslinked DNA from input and IP samples was released by incubating the eluate at 65°C overnight, and extracted with a solution of phenol/chloroform/isoamyl alcohol (25∶24∶1) prior to ethanol precipitation and resuspension in water. Mock IP reactions with no antibody yielded no measurable product (not shown) and were not quantified further.

ChIP-DNA for input and IP samples was end-repaired with Klenow DNA Polymerase and the DNA was purified with AMPure XP System beads (Beckman Coulter Genomics) and modified with A-tails using Klenow exo- before ligation to adapters to incorporate 7-base index sequences using T4 DNA ligase (Enzymatics). Adapter addition was confirmed on an Agilent 2100 bioanalyzer, and the DNA was PCR-amplified and then gel purified to remove adapter dimers and select sizes optimal for high-throughput sequencing (150 to 300 bp). Libraries were 12-way multiplexed on an individual lane of an Illumina Hi-Seq 2000 flow cell, resulting in approximately 7 million 42-bp single ended reads per sample.

Reads generated from the input and IP samples were aligned to the *C. neoformans* serotype A reference sequence [45] using Bowtie [67]. Reads that mapped to multiple genomic loci were discarded. Peak calling was performed using MACS [68] with a significance threshold of 1 × 10^-10^. To assess gross differences between the mutant and wild type, the average number of peaks over the three biological replicates of each strain was compared. Peaks were associated with specific genes if the peak center fell within 500 bp of the gene transcription start site according to the current annotation by the Broad Institute [45]. (For genes with unannotated 5′-UTRs this may correspond to the translation start site.) Ada2-dependent peak loss was identified by cases where a gene in two of the three wild type biological replicates possessed a neighboring peak and no peak was found to neighbor the gene in any of the three *ada2*Δ mutant replicates. ChIP-Seq data is available at GEO accession number GSE32075.

## Supporting Information

Figure S1 Ada2-dependent loss of H3-K9 acetylation is associated with activation. The ratio of Ada2 activated to Ada2 repressed genes (y-axis) is determined by an analysis of differential gene expression from RNA-Seq data comparing *ada2*Δ and wild type strains. The cutoff to be counted as differentially expressed is varied from 0 fold to ∼12 fold (x-axis). Ada2 activated genes exhibit a negative fold change greater than the cutoff and Ada2 repressed genes exhibit a positive fold change greater than the cutoff. Genes that lose neighboring H3-K9 acetylation near their TSS in the *ada2*Δ mutant are shown in red, genes that shown unchanged H3-K9 acetylation are shown in black.
(TIFF)

Table S1 Capsule-implicated genes. Genes for which the corresponding mutant strain has been shown to yield cells with altered capsule size or morphology.
(XLS)

Table S2 The transcriptional signature of capsule. Genes found to be significantly correlated with capsule size over panel of in vitro conditions are ordered by significance according to estimated false discovery rate (FDR).
(XLS)

Table S3 *C. neoformans* strains used in this study.
(DOC)

Table S4 Genes differentially expressed in the *ada2*Δ mutant. Genes found to be differentially expressed by RNA-Seq comparing wild type to *ada2*Δ mutant strains in either capsule inducing or non-inducing conditions (see Methods).
(XLS)

Table S5 Genes differentially expressed in the *nrg1*Δ mutant. Genes included are those found to be differentially expressed by RNA-Seq comparing wild type to *nrg1*Δ mutant strains in either capsule inducing or non-inducing conditions (see Methods).
(XLS)

Table S6 Genes differentially expressed in the *cir1*Δ mutant. Genes included are those found to be differentially expressed by RNA-Seq comparing wild type to *cir1*Δ mutant strains in either capsule inducing or non-inducing conditions (see Methods).
(XLS)

Table S7 Genes with neighboring histone acetylation peaks. Genes included are those with H3-K9 acetylation peaks within 500 bp of their transcription start site as currently annotated (see Methods). The number of biological replicates that support a neighboring peak is indicated for wild type and *ada2*Δ mutant strains.
(XLS)

Table S8 Primers used for constructing the mutant strains.
(DOC)

Table S9 Primers used to generate the *ADA2* reconstituted strain.
(DOC)

Table S10 Primers used to generate a strain expressing an HA epitope-tagged Ada2.
(DOC)

Text S1 Supplementary methods.
(DOC)
