# Supplementary figures and images for: Toward an Integrated Model of Capsule Regulation in Cryptococcus neoformans
Source: PLoS Pathog. 2011 Dec 8;7(12):e1002411. doi: 10.1371/journal.ppat.1002411 (PMC3234223; doi:10.1371/journal.ppat.1002411)

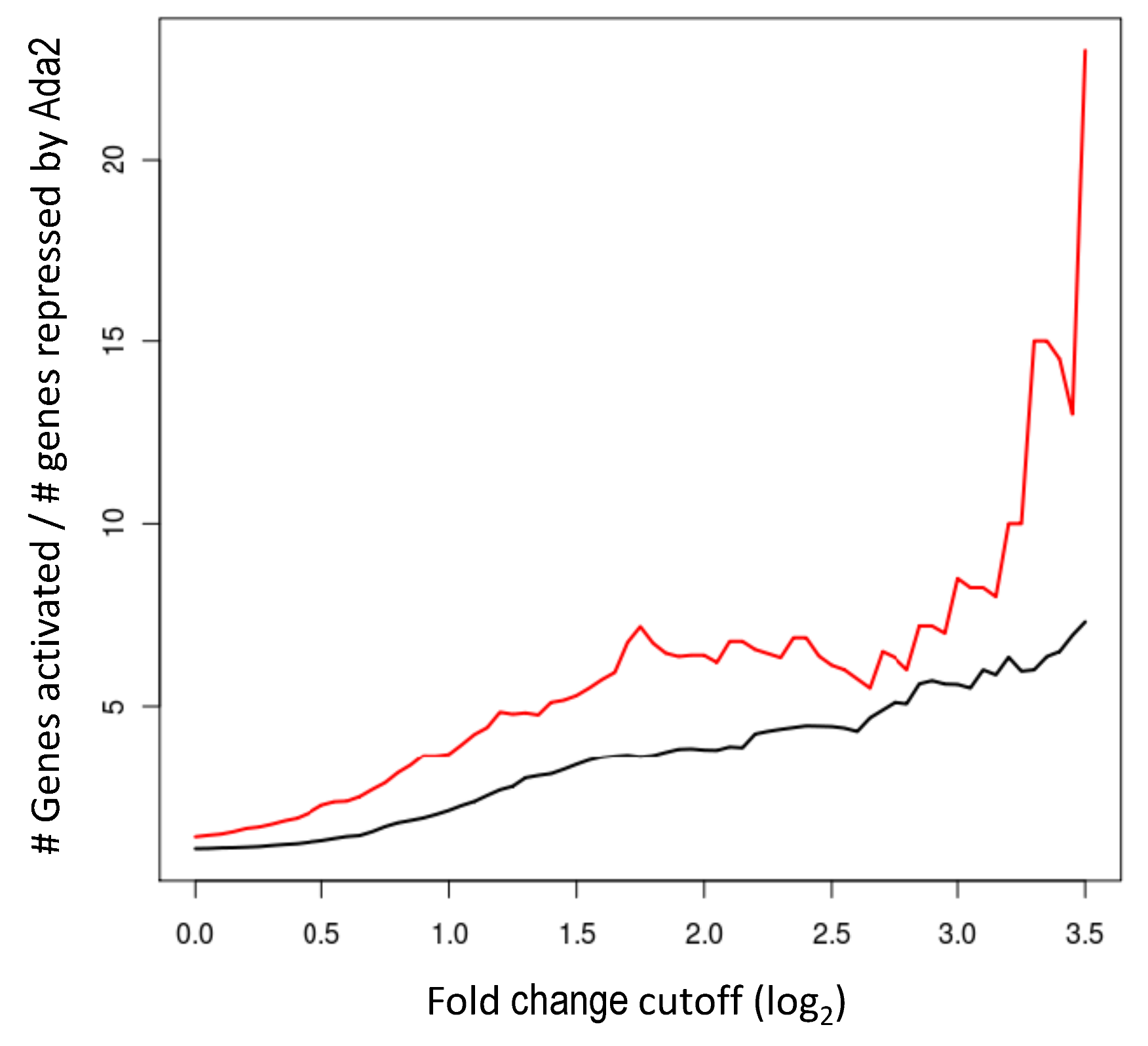

Supplement: Figure S1 — Ada2-dependent loss of H3-K9 acetylation is associated with activation. The ratio of Ada2 activated to Ada2 repressed genes (y-axis) is determined by an analysis of differential gene expression from RNA-Seq data comparing ada2Δ and wild type strains. The cutoff to be counted as differentially expressed is varied from 0 fold to ∼12 fold (x-axis). Ada2 activated genes exhibit a negative fold change greater than the cutoff and Ada2 repressed genes exhibit a positive fold change greater than the cutoff. Genes that lose neighboring H3-K9 acetylation near their TSS in the ada2Δ mutant are shown in red, genes that shown unchanged H3-K9 acetylation are shown in black. (TIFF) [file ppat.1002411.s001.tiff]
