# Supplementary material for: Toward an Integrated Model of Capsule Regulation in Cryptococcus neoformans
Source: PLoS Pathog. 2011 Dec 8;7(12):e1002411. doi: 10.1371/journal.ppat.1002411 (PMC3234223; doi:10.1371/journal.ppat.1002411)
Supplement: Table S3 — C. neoformans strains used in this study. (DOC) [file ppat.1002411.s004.doc]

| **Table S3. *C. neoformans* var. *grubii* strains used in this study.** | | |
| --- | --- | --- |
| **Strain** | **Additional information** | **Reference** |
| H99 | *MAT*α | Strain DP in [1] |
| KN99 | Derived from H99 | [2] |
| *ada2*∆^a^ | *ada2∆*::*NAT* ^b^ | This study |
| *ada2∆::ADA2* | *ada2∆*::*NAT* replaced with *ada2∆ ADA2-NEO* | This study |
| *ADA2-HA* | *ADA2* replaced with *ADA2*::*HA-NAT* | This study |
| *cap59*∆ | *cap59∆*::*HYG* | [3] |
| *cir1*∆ | *cir1∆*::*NAT* | This study |
| *nrg1*∆ | *nrg1∆*::*NAT* | This study |
| *ssn801*∆ | *ssn801∆*::*NEO* | This study |

^a^This and all strains below were derived from KN99α.

^b^*NAT* and *HYG* refer to cryptococcal nourseothricin and hygromycin resistance markers, respectively (see text and reference [3] below). *NEO* refers to a cryptococcal G418 resistance marker that, for the purposes of this study, consists of the bacterial neomycin phosphotransferase II (*nptII*) coding sequence under control of the cryptococcal *ACT1* promoter and *TRP1* terminator (i.e., P*_ACT1_*-*nptII*-T­*_TRP1_*).

1. Perfect JR, Lang SD, Durack DT. (1980) Chronic cryptococcal meningitis: a new experimental model in rabbits. Am J Pathol. 101(1):177-94.

2. Nielsen K, Cox GM, Wang P, Toffaletti DL, Perfect JR, Heitman J (2003) Sexual cycle of *Cryptococcus* *neoformans* var. *grubii* and virulence of congenic a and α isolates. Infect Immun 71: 4831-4841.

3. Baker LG, Specht CA, Donlin MJ, Lodge JK (2007) Chitosan, the deacetylated form of chitin, is necessary for cell wall integrity in *Cryptococcus neoformans*. Eukaryot Cell 6: 855-867.
