# Supplementary material for: Toward an Integrated Model of Capsule Regulation in Cryptococcus neoformans
Source: PLoS Pathog. 2011 Dec 8;7(12):e1002411. doi: 10.1371/journal.ppat.1002411 (PMC3234223; doi:10.1371/journal.ppat.1002411)
Supplement: Table S8 — Primers used for constructing the mutant strains. (DOC) [file ppat.1002411.s009.doc]

| **Table S8. Primers used for constructing the mutant strains.** | | | |
| --- | --- | --- | --- |
| **Primer name** | **Primer sequence (5’ to 3’) *** | **Comment** | **Primer direction** |
| MSPD-010 | GGATGCTCTCAGTGCATCCGC | *ADA2* - primer 1 | sense |
| MSPD-011 | gctctccagctcacatcctcgcagCGTGCCTCTTATTGCACGTC | *ADA2* - primer 2 | antisense |
| MSPD-013 | GAGAATCGTTGAACAGACCTC | *ADA2* - primer 3 | antisense |
| MSPD-014 | CgatagcgtcgaaagagacctcgtggacatcggGATTAGGCTCAATGGATGAGC | *ADA2* - primer 4 | sense |
| MSPD-127 | TGGCGTACTGTATCAATGTCAACCAAC | *CIR1* - primer 1 | sense |
| MSPD-129 | GACATATCAGCATAGAGACCTCGTGGACATCAACCATGCGTATGGGAACGGTG | *CIR1* - primer 2 | antisense |
| MSPD-130 | CAGGTACTGTGAATCTTGCTTGGGT | *CIR1* - primer 3 | antisense |
| MSPD-132 | gctctccagctcacatcctcgcagcGCTATGCACTATTAACAGAGAAGATGC | *CIR1* - primer 4 | sense |
| MSPD-076 | CAACCAATAGGAGACGCGTGTG | *NRG1* - primer 1 | sense |
| MSPD-077 | ccgTATGGCACGGCAGagagacctcgtggacatcGCAACTGTGGCTCAGCAAGAG | *NRG1* - primer 2 | antisense |
| MSPD-079 | GTGAGCATTACATATGCGCAGG | *NRG1* - primer 3 | antisense |
| MSPD-080 | gctctccagctcacatcctcgcagcGCAATGATGAGAGCCACCTG | *NRG1* - primer 4 | sense |
| AM-42 | CGCAACCTCAGAGCCAGATTG | *SSN801* - primer 1 | sense |
| AM-41 | CTATCGCATTCTGAGAGACCTCGTGGACATCCTAACTGCAGCTTTTATTGGTGTGTCT | *SSN801* - primer 2 | antisense |
| AM-45 | CCCAAGATCGACGTCATTGCTGG | *SSN801* - primer 3 | antisense |
| AM-44 | CGCTCTCCAGCTCACATCCTCGCAGCGGTCATGCCTTGATCATTTTCAAGTGC | *SSN801* - primer 4 | sense |
| MSPD-001 | gctgcgaggatgtgagctgg | *NAT* - primer 5 | sense |
| MSPD-034 | gctcatgtagagcgcctgctc | *NAT* - primer 6 | antisense |
| MSPD-033 | ccactcttgacgacacggcttacc | *NAT* - primer 7 | sense |
| AM-37 | GCTGCGAGGATGTGAGCTGG (same as MSPD-001) | *NEO* – primer 8 | sense |
| AM-54 | CACGGGTAGCCAACGCTATGTC | *NEO* – primer 9 | antisense |
| AM-55 | CCTGAATGAACTGCAGGACGAG | *NEO* – primer 10 | sense |
| MSPD-037 | ccGATGTCCACGAGGTCTCTTTCGACGCTATCGgctgcaggaattcgatatcaagc | *NAT* - primer 11 plus tag for *ADA2* | antisense |
| MSPD-094 | GATGTCCACGAGGTCTCTATGCTGATATGTCcgggctgcaggaattcgatatcaagc | *NAT* - primer 11 plus tag for *CIR1* | antisense |
| MSPD-095 | GATGTCCACGAGGTCTCTCTGCCGTGCCATAcgggctgcaggaattcgatatcaagc | *NAT* - primer 11 plus tag for *NRG1* | antisense |
| AM-40 | GATGTCCACGAGGTCTCTCAGAATGCGATAGCGGGCTGCAGGAATTCGATATC | *NEO* - primer 11 plus tag for *SSN801* | antisense |
| AM-63 | CCCCTTACCGCCTTCACGAATTCTCAGAAGAACTCGTCAAGAAGGCG | NEO- primer 12 | antisense |
| AM-64 | CGCCTTCTTGACGAGTTCTTCTGAGAATTCGTGAAGGCGGTAAGGGG | NEO-primer 13 | sense |

* The particular signature tag sequence used to label each deletion is given in blue type. The sequence of the priming site is in red type.
