# Supplementary material for: Toward an Integrated Model of Capsule Regulation in Cryptococcus neoformans
Source: PLoS Pathog. 2011 Dec 8;7(12):e1002411. doi: 10.1371/journal.ppat.1002411 (PMC3234223; doi:10.1371/journal.ppat.1002411)
Supplement: Table S9 — Primers used to generate the ADA2 reconstituted strain. (DOC) [file ppat.1002411.s010.doc]

| **Table S9. Primers used to generate the *ADA2* reconstituted strain.** | | | |
| --- | --- | --- | --- |
| **Primer name** | **Primer sequence (5’ to 3’)** | **Comment** | **Primer direction** |
| MSPD-001 | gctgcgaggatgtgagctgg | *NEO* 5' end | sense |
| MSPD-010 | GGATGCTCTCAGTGCATCCGC | *ADA2* upstream region | sense |
| MSPD-013 | GAGAATCGTTGAACAGACCTC | *ADA2* downstream region | antisense |
| MSPD-141 | tcctgcagcccGTGCCGAGGATTATGGGTCAAC | *ADA2* upstream region | antisense |
| MSPD-142 | CCCATAATCCTCGGCACgggctgcaggaattcgatatcaagc | *TRP1* terminator | antisense |
| MSPD-143 | gacgagttcttctgagaattcgtgaaggcggtaaggg | *TRP1* terminator | sense |
| MSPD-144 | cgccttcacgaattctcagaagaactcgtcaagaagg | *nptII* coding sequence | antisense |
| MSPD-145 | gctctccagctcacatcctcgcagcCGCATGAGATTAGGCTCAATGGA | *ADA2* downstream region | sense |
| MSPD-146 | cacgggtagccaacgctatgtc | *NEO* 5' end | antisense |
| MSPD-147 | cctgaatgaactgcaggacgag | *nptII* coding sequence | sense |
