# Supplementary material for: Toward an Integrated Model of Capsule Regulation in Cryptococcus neoformans
Source: PLoS Pathog. 2011 Dec 8;7(12):e1002411. doi: 10.1371/journal.ppat.1002411 (PMC3234223; doi:10.1371/journal.ppat.1002411)
Supplement: Table S10 — Primers used to generate a strain expressing an HA epitope-tagged Ada2. (DOC) [file ppat.1002411.s011.doc]

| **Table S10. Primers used to generate a strain expressing an HA epitope-tagged Ada2.** | | | |
| --- | --- | --- | --- |
| **Primer name** | **Primer sequence (5’ to 3’)** | **Comment** | **Primer direction** |
| LH-201 | CAAACCTCGTGGGTGTGATG | *ADA2* upstream region | sense |
| LH-202 | cctaggtcaagcgtaatcagggacatcgtaagggtaTCCATTGAGCCTAATCTCATGCG | *ADA2* upstream region | antisense |
| LH-203 | gtccctgattacgcttgacctaggGCAAATTTATAGTCACTATTCTTCAAAAAG | *ADA2* terminator | sense |
| LH-204 | ctccagctcacatcctcgcagccctgcaggAAGCATCCCATGGGAGTCAG | *ADA2* terminator | antisense |
| LH-205 | gtttctacatctcttctataagcttgcctgcaggATCATGCGATTGTGTTGTTG | *ADA2* downstream region | sense |
| LH-206 | GACAGAGCTGCCGATGTTAC | *ADA2* downstream region | antisense |
| LH-231 | gctgcgaggatgtgagctg | *NAT* 5' end | sense |
| LH-034 | gctcatgtagagcgcctgctc | *NAT* 5' end | antisense |
| LH-035 | gccactcttgacgacacggcttac | *NAT* 3' end | sense |
| LH-232 | caagcttatagaagagatgtagaaactagcttcc | *NAT* 3' end | antisense |
