# Supplementary material for: Toward an Integrated Model of Capsule Regulation in Cryptococcus neoformans
Source: PLoS Pathog. 2011 Dec 8;7(12):e1002411. doi: 10.1371/journal.ppat.1002411 (PMC3234223; doi:10.1371/journal.ppat.1002411)
Supplement: Text S1 — Supplementary methods. (DOC) [file ppat.1002411.s012.doc]

Toward an integrated model of capsule regulation in *Cryptococcus neoformans*

Haynes, Skowyra, *et al*

**SUPPLEMENTARY METHODS**

Construction of deletion strains:

To assemble the split-marker cassettes, the region upstream of the translation start site of each target gene was first amplified from KN99α genomic DNA by PCR using primers 1 and 2 (all gene-specific primers in this section are listed in Table S7). Similarly, the region downstream of the translation stop site of each gene was amplified using primers 3 and 4. Next, the 5' region of *NAT* was amplified from plasmid pGMC200 [1] using primers 5 and 6; likewise, the 3' region of the marker was amplified from the same plasmid using primer 7 and the appropriate version of primer 11. Similarly, the 5‘ region of the G418 resistance marker (NEO) was amplified from plasmid pMH12T [2] using primers 8 and 9; the 3‘ region of the marker was amplified using primer 10 and the appropriate primer 12. The TRP1 terminator was amplified using primers 11 and 13. The terminator was fused to the 3‘ end of the G418 resistance marker using primers 10 and 11. For generating the *ada2∆* mutants, the region immediately upstream of the *ADA2* genomic coding sequence was fused to the 5' portion of *NAT* by including both pieces as templates in one PCR with primers 1 and 6. Similarly, the region immediately downstream of the *ADA2* genomic coding sequence was fused to the corresponding 3' end of the marker using primers 4 and 7. For generating the *cir1∆*, *ssn801∆*, and *nrg1∆* mutants, marker orientation was reversed, so that the upstream flanking region of each gene was fused to the 3' portion of *NAT or NEO* using primers 1 and 7 or 1 and _9_ respectively, and the downstream region was fused to the 5' portion of the marker using primers 4 and 6 or 3 and _10_ respectively. Each corresponding pair of split-marker cassettes was co-precipitated onto gold beads and used to biolistically transform KN99α cells as described [3]. Nourseothricin-resistant or G418-resistant colonies were restreaked twice on YPD plates, and two independently-generated transformants with the desired gene replacement (assessed by PCR and DNA blotting, data not shown) were maintained. The *ada2*∆ mutants were backcrossed sequentially to parental KN99α and KN99**a** strains for isolation of congenic MATα and MAT**a** mutant strains as described [4]. Progeny strains from each transformant were comparable in all phenotypic analyses (data not shown).

We also used a split marker approach to complement one *ada2*∆ strain by replacing the *NAT* marker with the *ADA2* genomic coding sequence fused to a different marker. To assemble one of the split-marker pieces, we used primers MSPD-010 and MSPD-141 (Table S8) to PCR-amplify the *ADA2* coding sequence from KN99α genomic DNA along with 1.2 kb of upstream and 279 bp of downstream sequence. The *TRP1* terminator was amplified from plasmid pGMC200 using primers MSPD-142 and MSPD-143, and the 3' end of the neomycin phosphotransferase II (*nptII*) coding sequence was amplified from the G418 resistance marker (*NEO*) on plasmid pMH12T [2] using primers MSPD-144 and MSPD-147. The *TRP1* terminator was first fused to the truncated *nptII* coding sequence by including both pieces as templates in one PCR with primers MSPD-142 and MSPD-147; in a second round of PCR, the resulting amplicon was fused to the 3‘ end of the *ADA2* coding fragment using primers MSPD-010 and MSPD-147. The assembled construct was TA-cloned into pCR-XL-TOPO (from Invitrogen), the *ADA2* coding sequence confirmed by DNA sequencing, and the construct subsequently released using SpeI and NotI. To assemble the second split-marker construct, the 5' end of *NEO* was first amplified using primers MSPD-001 and MSPD-146. Next, a region begining 25 bp upstream of the *ADA2* translation stop site and extending 1.2-kb downstream was amplified from KN99α genomic DNA using primers MSPD-145 and MSPD-013. This piece was then fused to the 5' end of *NEO* by PCR using primers MSPD-146 and MSPD-013. The split-marker cassettes were co-precipitated onto gold beads and used to biolistically transform one *ada2*∆ mutant as described above. Colonies that were both G418-resistant and nourseothricin-sensitive were restreaked twice on YPD, and several independently-generated transformants with the correctly reintegrated *ADA2* coding sequence (assessed by PCR) were maintained.

Expression of epitope-tagged Ada2.

We used the split-marker gene replacement approach described above to introduce a single copy of the hemagglutinin (HA) epitope-tag sequence at the 3' end of the *ADA2* genomic coding sequence. To assemble the first split-marker construct, a 500-bp region immediately upstream of the *ADA2* translation stop site was amplified from KN99α genomic DNA by PCR using primers LH-201 and LH-202 (Table S9). A 200-bp region immediately downstream of the translation stop site was amplified using primers LH-203 and LH-204, and the 5' end of *NAT* was amplified from plasmid pGMC200 using primers LH-231 and LH-034. All three pieces were combined in one PCR using primers LH-201 and LH-034. The resulting amplicon was TA-cloned into pCR2.1-TOPO (from Invitrogen), confirmed by DNA sequencing, and subsequently released using SpeI and XbaI. To assemble the second split-marker construct, the 3' end of *NAT* was first amplified from plasmid pGMC200 using primers LH-035 and LH-232. Next, a 292-bp region beginning 200 bp downstream of the *ADA2* translation stop site was amplified from KN99α genomic DNA using primers LH-205 and LH-206, and fused to the 3' end of the marker by PCR using primers LH-035 and LH-206. The two split-marker constructs were gel-purified, co-precipitated onto gold beads, and used to biolistically transform KN99α cells as described above. Nourseothricin-resistant colonies were restreaked twice on YPD, correct integration of the epitope-tag sequence was confirmed by PCR, and expression of the expected size polypeptide was confirmed by immunoblotting.

Phenotypic testing.

To test mutant growth under stress conditions, cells cultured overnight in YPD were collected by centrifugation, adjusted to 2 × 10^7^ cells/ml in PBS, and three 10-fold serial dilutions were prepared. To test oxidative and nitrosative stress sensitivity, 5-µl aliquots of the original cell suspension and each dilution were spotted onto solid YNB medium (0.67% w/v yeast nitrogen base without amino acids, 2% w/v glucose, 2% w/v agar, 25 mM sodium succinate pH 4.0) containing either 0.5 mM hydrogen peroxide (H_2_O_2_) or 0.5 mM sodium nitrite (NaNO_2_), respectively. For other studies, 5-µl aliquots were spotted onto solid YPD medium with or without the following compounds: 50 mM MES pH 5.5; 50 mM Tris pH 8.8; 0.4 M or 1.2 M NaCl; 0.4 M or 1.2 M KCl; 0.4 M CaCl_2_; 150 mM LiCl; 6% v/v ethanol; 0.01% (w/v) sodium dodecyl sulfate (SDS); 0.2% (w/v) Calcofluor white (Fluorescent Brightener 28; from Sigma); 0.5% (w/v) Congo Red (from Sigma); or 15 mM caffeine. Melanization was assessed on agar plates containing 1 mM L-3,4-dihydroxyphenylalanine (L-DOPA) as described [5]. All plates were incubated at 30ºC, 37ºC, or 39ºC, as indicated in the text.

Susceptibility to amphotericin B, fluconazole, and 5-fluorocytosine was determined using Sensititre YeastOne standard susceptibility plates (from TREK Diagnostic Systems) as specified by the manufacturer. Susceptibility to FK-506 was performed as described [6]. Briefly, cells were grown overnight in YPD and adjusted to 10^5^ cells/ml with YPD. A 5 mg/ml solution of FK-506 was prepared in DMSO, and serially diluted in YPD to the following concentrations: 2, 1, 0.5, 0.25, 0.125, 0.06, 0.03 µg/ml. A 10-µl aliquot of each cell suspension was added to 100 µl of each FK-506 solution or YPD alone in a 96-well microtiter plate, the plates were incubated for 48 h at 30°C with agitation, and absorbance at 600 nm was measured as an indicator of cell growth.

References

1. McDade HC, Cox GM (2001) A new dominant selectable marker for use in *Cryptococcus neoformans*. Medical Mycology 39: 151-154.

2. Hua J, Meyer JD, Lodge JK (2000) Development of positive selectable markers for the fungal pathogen *Cryptococcus neoformans*. Clinical and Diagnostic Laboratory Immunology 7: 125-128.

3. Toffaletti DL, Rude TH, Johnston SA, Durack DT, Perfect JR (1993) Gene transfer in *Cryptococcus neoformans* by use of biolistic delivery of DNA. Journal of Bacteriology 175: 1405-1411.

4. Nielsen K, Cox GM, Wang P, Toffaletti DL, Perfect JR, et al. (2003) Sexual cycle of *Cryptococcus neoformans var. grubii* and virulence of congenic **a** and alpha isolates. Infection and Immunity 71: 4831-41.

5. Eisenman HC, Mues M, Weber SE, Frases S, Chaskes S, et al. (2007) *Cryptococcus neoformans* laccase catalyses melanin synthesis from both D- and L-DOPA. Microbiology (Reading, England) 153: 3954-62.

6. O`Meara TR, Hay C, Price MS, Giles S, Alspaugh JA (2010) *Cryptococcus neoformans* histone acetyltransferase Gcn5 regulates fungal adaptation to the host. Eukaryotic Cell 9: 1193-202.
